# Single nucleus multiomics reveals an early inflammatory response to high-fat diet in mouse islets

**DOI:** 10.26508/lsa.202603840

**Published:** 2026-09-01

**Authors:** Isabell VS Ernst, Lasse Lehtonen, Freja L Nielsen, Morten F Ebbesen, Sille M Nilsson, Ann-Britt Marcher, Susanne Mandrup, Jesper GS Madsen

**Affiliations:** 1 Functional Genomics and Metabolism Research Unit, Department of Biochemistry and Molecular Biology, University of Southern Denmark , Odense, Denmark; 2 Danish Molecular Biomedical Imaging Center (DaMBIC), Department of Biochemistry and Molecular Biology, University of Southern Denmark , Odense M, Denmark; 3 Center for Functional Genomics and Tissue Plasticity (ATLAS), Odense M, Denmark; 4 Center for Adipocyte Signaling (ADIPOSIGN), Odense M, Denmark; 5 The Novo Nordisk Foundation Center for Genomic Mechanisms of Disease, Broad Institute of MIT and Harvard, Cambridge, MA, USA

## Abstract

In periods of sustained hyper-nutrition, pancreatic β-cells undergo functional compensation through transcriptional upregulation of gene programs driving insulin secretion. This adaptation is essential for maintaining systemic glucose homeostasis and metabolic health. Using single nuclei multiomics, we have mapped the early transcriptional adaptive mechanisms in murine islets of Langerhans exposed to high-fat diet (HFD) for 1 and 3 wk. We show that β-cells exhibit the largest transcriptional response to HFD, characterized by early activation of pro-inflammatory eRegulons and down-regulation of β-cell identity genes, particularly in a distinct subset of β-cells. These observations extend to humans, where the prevalence of an β-cells with a high inflammatory signature is increased in diabetes. Collectively, these observations point to cellular crosstalk through pro-inflammatory signaling as a central and early driver of β-cell dysfunction that limits the compensatory capacity of β-cells, which is closely linked to the development of diabetes.

## Abstract (precis)

Single-nucleus multiomics identifies a β-cell subpopulation mounting an early interferon-driven inflammatory response to high-fat diet, with traces of this signature detected in human diabetes.

## Introduction

The increasing incidence of type 2 diabetes mellitus (T2DM), which coincides with a growing prevalence of obesity and a sedentary lifestyle (1, 2), constitutes a major global health threat. T2DM is a pleiotropic disease, which involves both decreased responsiveness to insulin in multiple tissues and inadequate insulin secretion by pancreatic β-cells. Thus, the ability of the β-cells to adapt to increased nutrient intake and secrete adequate amounts of insulin plays a key role in preventing the development of T2DM.

Functional compensation to meet the increased demand for insulin involves both increased β-cell mass and upregulation of insulin secretory capacity. Cellular adaptation through transcriptional and metabolic regulation plays a crucial role during functional compensation, where β-cells become more sensitive to glucose, exhibit enhanced glucose uptake and metabolism, and upregulate pathways involved in the production and secretion of insulin (3). In addition, excess nutrients, such as glucose and free fatty acids are directed away from harmful metabolic processes through storage, conversion, or export, which enables the β-cell to maintain function (4). The loss of the ability to compensate represents a crucial tipping point in the development of T2DM, and understanding the underlying mechanisms is essential to understanding T2DM.

Single-cell technologies have shed light on the functional and transcriptional heterogeneity of β-cells. For example, several single-cell RNA-seq (scRNA-seq) studies have observed heterogeneity in the functional maturity of β-cells in islets from adult humans and mice (5, 6, 7). Specifically, it has been suggested that adult human β-cells exist in at least two or three different states (6, 8, 9, 10, 11, 12, 13) which differ in terms of their epigenome, transcriptome, and electrophysiology. Interestingly, studies have observed a shift in the relative abundance of the β-cell subpopulations during development of T2DM. Machine learning analysis of single nucleus chromatin accessibility in islets from nondiabetic, prediabetes, and T2DM donors, identified two functionally distinct β-cell subtypes that shift in abundance during T2DM progression (8). Another study found that the distribution of four antigenically identified β-cell subtypes, all present in nondiabetic individuals, was altered in individuals with T2DM (10), suggesting that control of β-cell function is at least partly regulated through cell state transition.

To investigate the dynamic gene-regulatory mechanisms controlling β-cell transitions during adaptation to the high-fat diet (HFD) challenge, we fed mice a HFD for 1 and 3 wk. We used single-nucleus multi-omics to simultaneously analyze changes in chromatin accessibility using single-nucleus assay for transposase-accessible chromatin using sequencing (snATAC-seq) and gene expression using single-nucleus RNA-seq (snRNA-seq) in islets of Langerhans. Here, we show that of the different cell types in the islets, β-cells are most strongly affected by short-term HFD. We map gene regulatory networks and discover a subpopulation of β-cells that are marked by pro-inflammatory gene signatures that correlate to systemic metabolic traits in mice. This gene signature is also detected in β-cells from humans who had prediabetes or diabetes, suggesting that inflammation may be an important regulator of the capacity for functional compensation in β-cells.

## Results

### Characterization of islets of Langerhans during short-term high-fat diet

**Figure 1.**
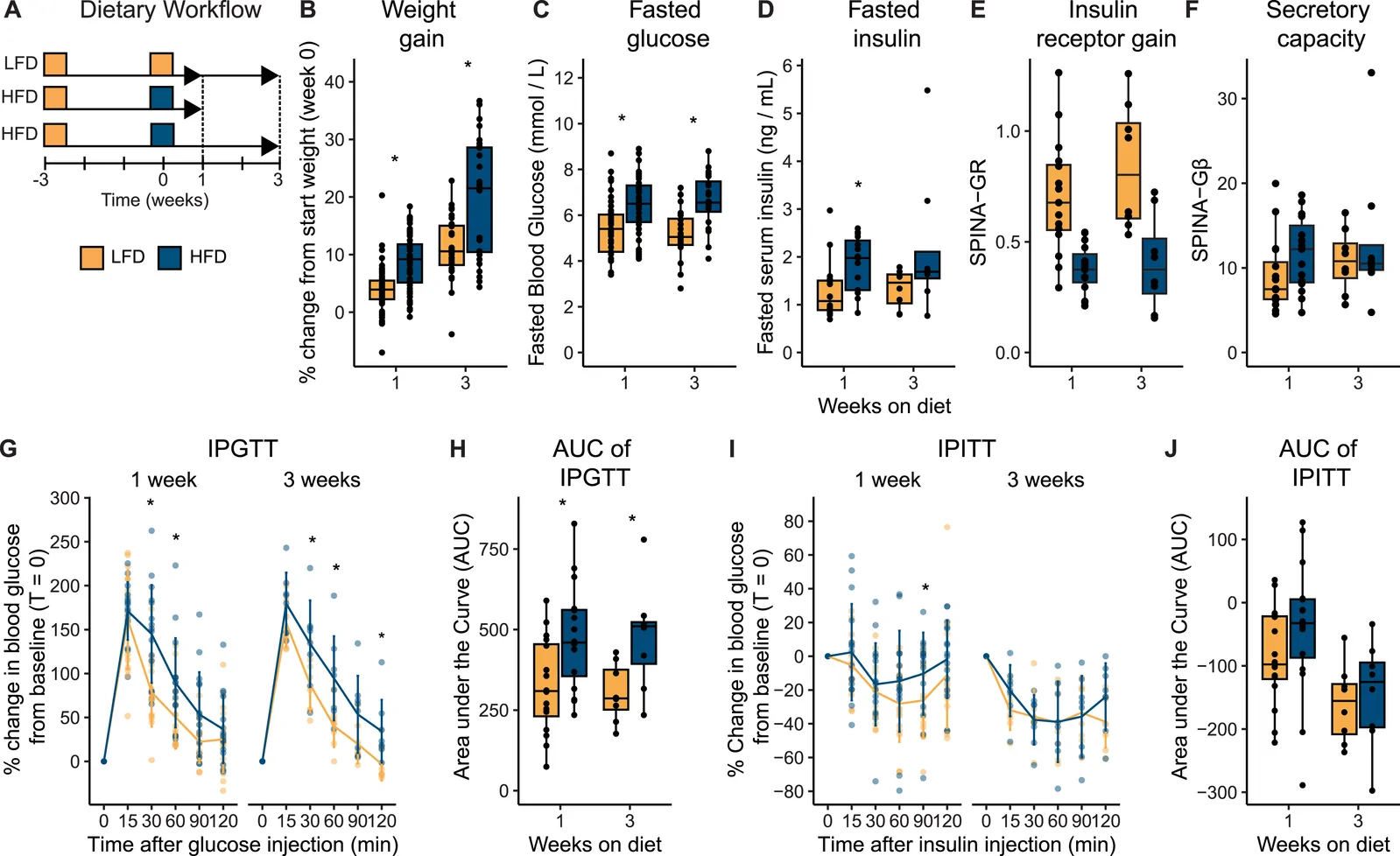
Short-term high-fat diet leads to increased body weight gain, fasting hyperglycemia, hyperinsulinemia, mild glucose intolerance, and onset of insulin resistance. **(A)** Schematic overview of the experimental setup: 8-wk-old male C57BL/6JBomTac mice underwent a 3-wk acclimatization period during which they were fed a low-fat diet (LFD). Subsequently, mice were divided into groups receiving either LFD (orange, as a control) or high-fat diet (blue, HFD) for 1 or 3 wk. **(B)** Weight gain measured as a percentage of starting weight at day 0 for each animal after 1 wk (n = 66–70 per diet) and 3 wk (n = 28 per diet). **(C)** Fasting blood glucose levels in mmol/liter at 1 wk (n = 48 per diet) and 3 wk (n = 24 per diet). **(D)** Fasting serum insulin levels in ng/ml at 1 wk (n = 15–16 per diet) and 3 wk (n = 8 per diet). **(E)** Structure parameter inference approach (SPINA)-Carb of insulin receptor gain/insulin sensitivity (SPINA-GR) at 1 wk (n = 15–16 per diet) and 3 wk (n = 8 per diet). **(F)** Structure parameter inference approach (SPINA)-Carb of secretory capacity/β-cell function (SPINA-Gβ) at 1 wk (n = 15–16 per diet) and 3 wk (n = 8 per diet). **(G)** Intraperitoneal glucose tolerance test (IPGTT) at 1 wk (n = 16 per diet) and 3 wk (n = 8 per diet). Data is shown as a percentage change from blood glucose at T = 0. **(H)** The data points shown in the IPGTT (G) were used to calculate the area under the curve (AUC). **(I)** Intraperitoneal insulin tolerance test (IPITT) at 1 wk (n = 16 per diet) and 3 wk (n = 8 per diet). Data are shown as a percentage change from serum insulin levels at T = 0. **(J)** The data points shown in the IPITT (I) were used to calculate the AUC. Asterisk indicates FDR-adjusted *P*-value ≤0.05. Source data are available for this figure.

**Figure S1.**
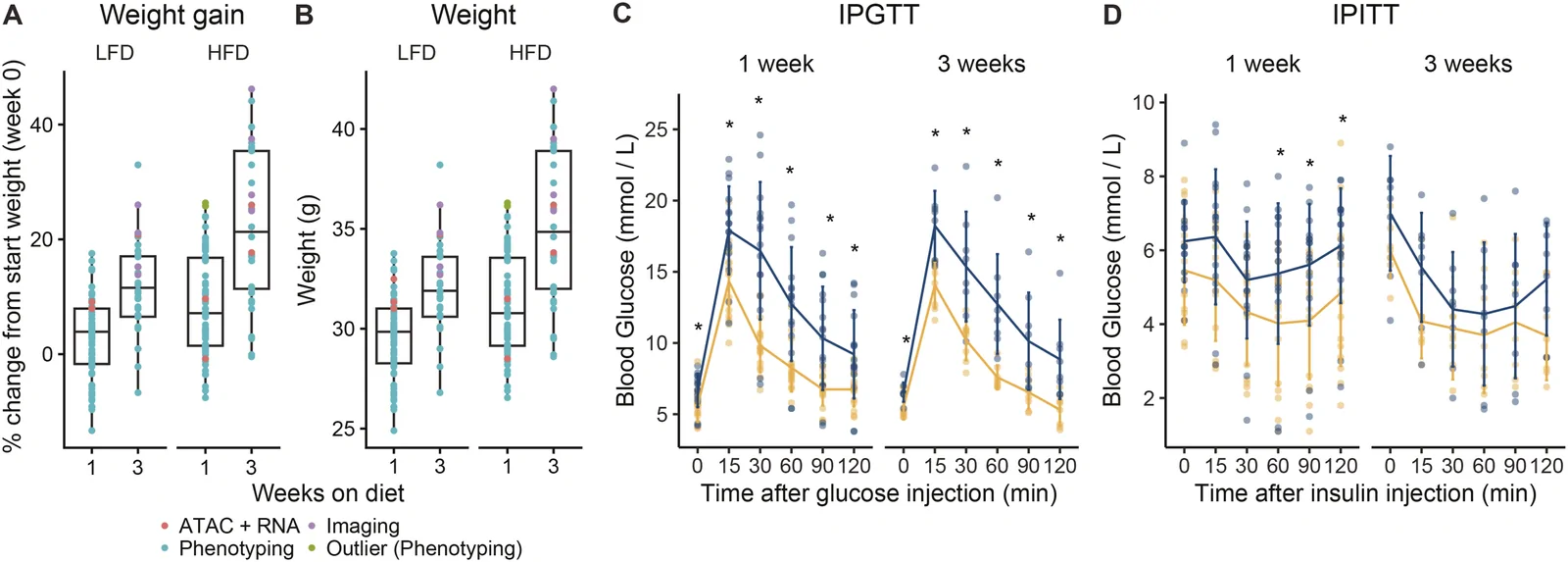
Related to Fig 1. 11-wk-old male C57BL/6JBomTac mice were fed either a low-fat diet (LFD, orange) or a high-fat diet (HFD, blue) for 1 and 3 wk. **(A)** Weight gain measured as a percentage change from baseline weight (week 0) and absolute body weight. **(B)** in mice fed LFD or HFD (n = 69–72 per diet) used for the phenotyping experiment at 1 and 3 wk, as well as mice used for Single Cell Multiome ATAC + Gene expression sequencing (ATAC+RNA; LFD: n = 2 at 1 wk, n = 1 at 3 wk; HFD: n = 2 at both timepoints), and imaging analyses (n = 4 per diet). **(C)** Fasted Intraperitoneal Glucose Tolerance Test (GTT) at 1 wk (n = 16 per diet) and 3 wk (n = 8 per diet). **(D)** Fasted Intraperitoneal Insulin Tolerance Test (ITT) at 1 wk (n = 16 per diet) and 3 wk (n = 8 per diet). Points represent individual animals. Boxplots show the median and interquartile range. Asterisk indicates FDR-adjusted *P*-value ≤0.05.

To gain cell type-resolved insight into the transcriptional events regulating the adaption of islets of Langerhans, we initially established and characterized an experimental setup using short-term HFD feeding in mice. 8-wk-old male C57BL/6JBomTac mice were first exposed to low-fat diet (LFD) for 3 wk, whereafter they were either maintained on the LFD (control) or switched to HFD for 1 or 3 wk (Fig 1A and Table S1). HFD-fed mice exhibited increased body weight gain, fasting hyperglycemia, hyperinsulinemia, mild glucose intolerance, and onset of insulin resistance compared with LFD controls already after 1 wk (Figs 1B–J, and S1A–D), and these phenotypic differences between HFD and LFD-fed mice were maintained after 3 wk of HFD, except for a more pronounced increase in body weight. These results are consistent with previous studies (14, 15, 16, 17, 18) and indicate that the 1 and 3 wk-time points are adequate for inducing a compensatory response. In line with these observations, the secretory capacity of pancreatic β-cells, estimated by the structure parameter inference approach (SPINA-Gβ), increased after 1 wk of HFD compared with LFD controls, although this difference did not reach statistical significance (*P* = 0.065). SPINA-Gβ is a model-based index calculated from fasting glucose and insulin that reflects β-cell secretory capacity at steady state (19). It does not capture stimulus-driven insulin secretion during a glucose challenge but rather provides a fasting surrogate of baseline β-cell capacity. Within this limitation, our data suggests that after 1 wk of HFD, β-cell compensation has been induced.

Table S1. Diet composition.

**Figure S2.**
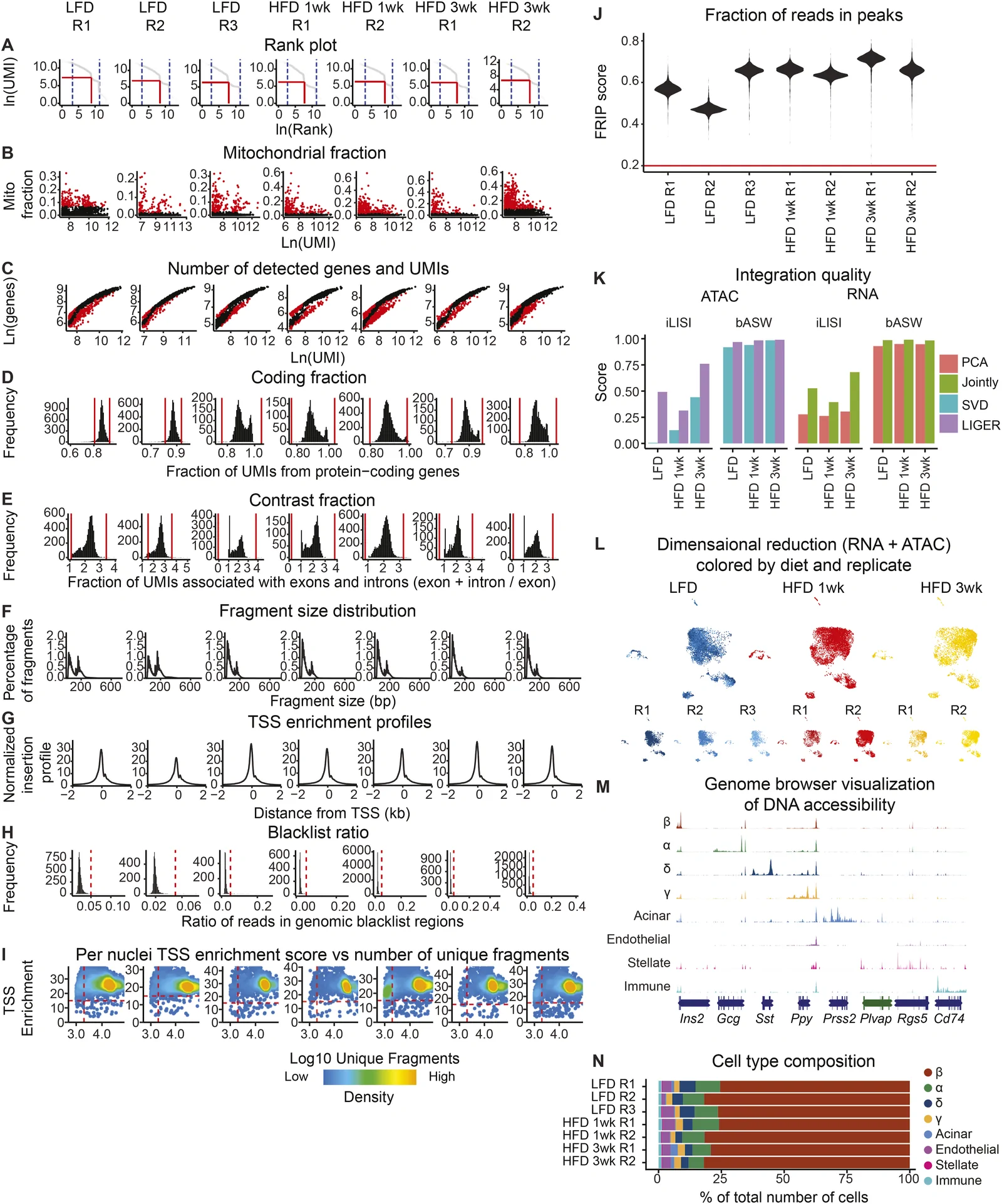
Related to Fig 2. **(A)** Rank plot of log-transformed total number of unique molecular identifiers (UMIs) and the log-transformed rank in order of decreasing total UMIs for the indicated sample. Vertical red line indicates the threshold, and the red horizontal line indicates the corresponding number of UMIs. The dashed blue vertical lines represent the quantile range for the breakpoint search. These lines define the region within which breakpoints are searched. **(B)** Scatter plot of the log-transformed number of detected features and the fraction of UMIs associated with mitochondrially encoded genes. Red nuclei were filtered away. **(C)** Scatter plot showing the log-transformed total number of UMIs and the number of detected features. The blue line shows piecewise linear regression with three breakpoints. Red nuclei were filtered away. **(D)** Histogram showing the distribution of UMIs associated with protein-coding genes. Nuclei falling below or above the red lines were removed. **(E)** Histogram showing the distribution of the fraction of UMIs derived from exons (contrast fraction). Nuclei with a contrast fraction below 1 were removed. **(F)** Fragment size distribution plot. **(G)** Mean Tnf5 insertion frequency around transcription start sites (TSS). **(H)** Histogram showing the distribution of the fraction of reads in blacklist regions (blacklist ratio). Nuclei falling above the red line were removed. **(I)** Scatter plot showing TSS enrichment score and log10 number of unique fragments. Nuclei falling below the black lines were removed. **(J)** Violin plot of the fraction of reads within peaks (FRiP) for each replicate. Nuclei falling below the red line were removed. **(K)** Bar plot depicting integration quality based on the ATAC data and RNA data. The global integration local Inverse Simpson’s Index (iLISI) and the batch average silhouette width (bASW) scores in SVD, LIGER, PCA, and JOINTLY were calculated for each condition (x-axis). **(L)** Uniform Manifold Approximation and Projection (UMAP) visualization of clustering based on a weighted nearest neighbor graph, based on the weighted average of RNA and ATAC similarities, split by cluster and replicate. Nuclei are colored by condition and replicate. **(M)** Pseudo-bulk chromatin accessibility tracks scaled between 0–1 for endocrine marker genes by each cell type. **(N)** Bar plot depicting the percentage of cells in each cell type per condition for each replicate.

**Figure 2.**
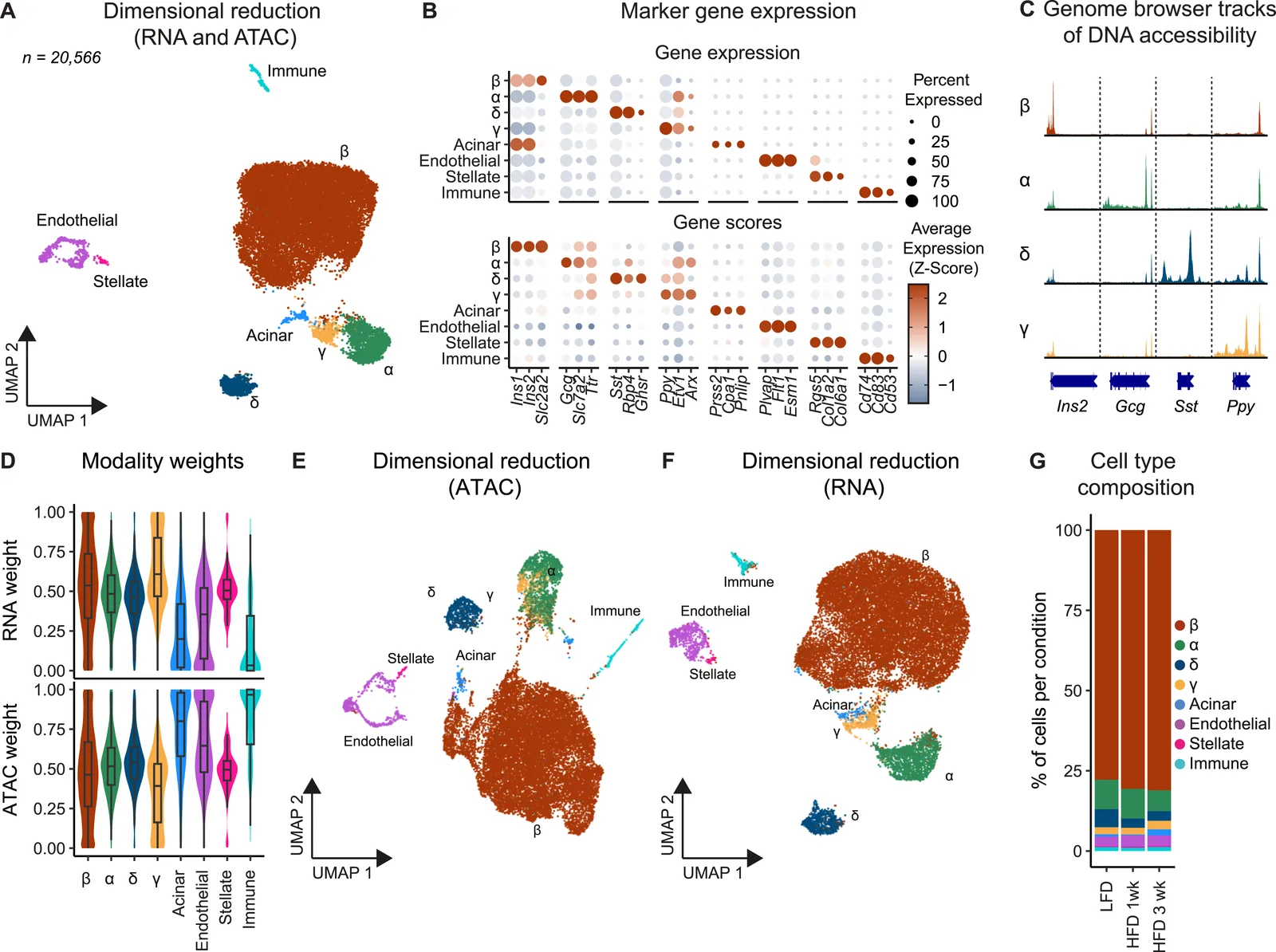
Classification of islet cell types through integrated snRNA and snATAC-seq analyses. **(A)** 11-wk-old male C57BL/6JBomTac mice were fed either a low-fat diet (LFD) (n = 3) or high-fat diet (HFD) for one (1 wk) (n = 2) and three (3 wk) (n = 2) weeks. At the end of the experiment, the mice were euthanized, and islets of Langerhans were isolated. Nuclei from islets were purified and used for Single Cell Multiome ATAC + Gene expression sequencing. The scatterplot shows Uniform Manifold Approximation and Projection (UMAP) visualization of clustering based on a weighted nearest neighbor (WNN) graph, based on the weighted average of RNA and ATAC similarities (n = 20,566). Data points are colored by cell type as indicated on the figure. **(B)** Dot plot showing the percentage of cells expressing the gene (dot size) and scaled average gene expression (dot color) of cell type marker genes (21) per cell type in the top panel, as well as the average gene score, calculated based on the chromatin accessibility of regulatory elements in the vicinity of the gene, of cell type markers in the bottom panel. **(C)** Pseudo-bulk chromatin accessibility tracks scaled between 0–1 for endocrine marker genes by each endocrine cell type. **(D)** Violin and boxplot depicting the distribution of the per-nucleus RNA (top) and ATAC (bottom) modality weights, divided by cell type. **(E, F)** UMAP visualization of clustering based on the ATAC (E) or RNA (F) modality. **(G)** Staged bar plot depicting the percentage (%) of cells in each cell type per condition. Source data are available for this figure.

To investigate the single cell-resolved transcriptional response of islets to HFD, we jointly measured both chromatin accessibility and gene expression using snATAC-seq and snRNA-seq, respectively, in single nuclei from isolated islets of Langerhans from mice fed with LFD for 1 (n = 2) or 3 wk (n = 1), or HFD for 1 (n = 2) or 3 wk (n = 2). Following rigorous quality filtering of both the snRNA-seq and snATAC-seq data (Fig S2A–J and Table S2), a total of 20,566 nuclei were included in the downstream analyses. We performed dimensional reduction and batch integration for each modality (Fig S2K). Using weighted nearest neighbor (WNN) analysis (20), we created a joint embedding incorporating information from both data modalities and used it for unsupervised clustering (Figs 2A and S2L). We merged and labeled the clusters as eight different cell types based on marker gene expression, predicted gene scores, and pseudo-bulk chromatin accessibility tracks of known major marker genes in each cluster (Figs 2B and C and S2M). Both data modalities contributed equally to identifying the endocrine cell type populations based on modality-specific weights (Fig 2D), except for γ-cells, which were masked in the snATAC-seq data (Fig 2E) but not in the snRNA-seq data (Fig 2F).

Table S2. Islet and nuclei isolation summary of sequencing samples.

The relative contribution of the identified cell types were similarly represented across conditions (Figs 2G and S2N) and correspond to the expected major cell types in islets in concordance with the literature (22) with β-cells comprising the largest proportion of the endocrine cells (∼78–81%), followed by α-cells (∼7–9%), δ-cells (∼3–6%), and γ-cells (∼2–3%). In addition, we identified a small subset of endothelial cells, stellate cells and immune cells (∼3%, ∼0.3% and ∼1%, respectively), and only a low percentage of acinar cells (∼0.3–2%), indicating an overall high purity of the islet preparations with minimal exocrine contamination. The fraction of β-cells trends toward being increased with HFD feeding at the expense of α-cells and δ-cells.

### Short-term high-fat diet induces a pro-inflammatory response in β-cells

**Figure 3.**
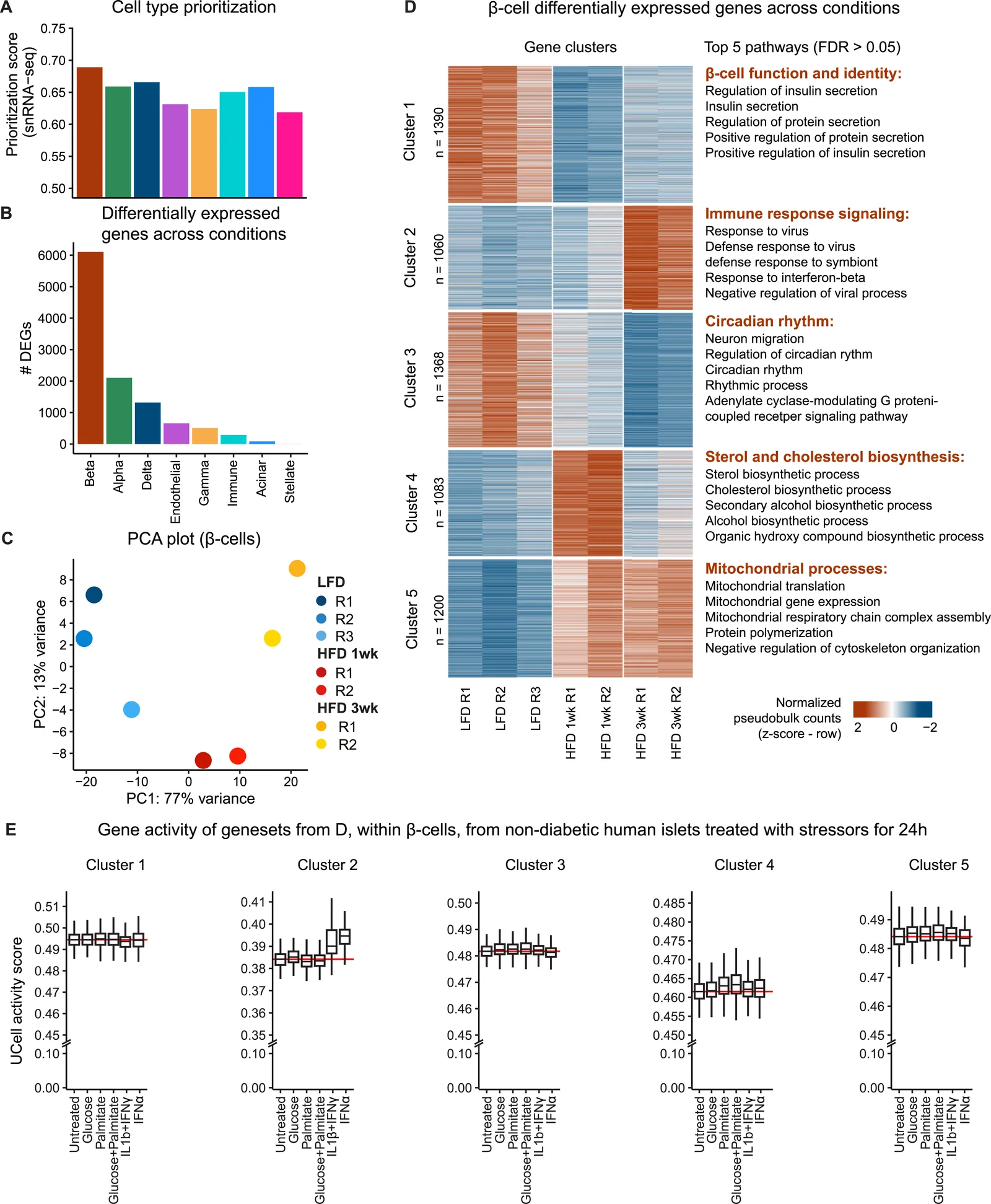
Transcriptional dynamics of β-Cells in response to 1 and 3 wk of high-fat diet. **(A)** Bar plot of Augur cell type prioritization scores across conditions, based on single-nucleus (sn)RNA-seq data, identified for each cell type. **(B)** Bar plot of the number of differentially expressed genes identified for each cell type. **(C)** Principal component analysis plot from the first two principal components calculated from pseudo-bulk snRNA-seq data from β-cells across all replicates, using the top 500 most variable genes. Each point represents a sample, with color and shading according to diet and replicate. **(D)** Left panel: Heatmap showing z-transformed normalized pseudo-bulk average expression of differentially expressed genes identified within β-cells. Genes were divided into five clusters using fuzzy c-means clustering. Right panel: The top five most significant pathways from Gene Ontology biological processes within each fuzzy cluster. **(E)** Boxplot of the per cell gene activity score of gene clusters from Fig 3D, within pancreatic β-cells from non-diabetic human donors which have been separated into groups treated with stressors; Glucose (22 mM) and/or palmitate (0.5 mM), interleukin-1beta (IL-1β) (1 ng/ml), and interferon gamma (IFNγ) (1,000 U/ml), or interferon alpha (IFNα) (2,000 U/ml) compared with untreated controls for 24 h (24, 25). Red lines indicate the median in the untreated samples. Number of donors in each condition at each time point: 24 h; Glucose (n = 3), palmitate (n = 3), Glucose + Palmitate = (n = 3), IL-1β and IFNγ (n = 3), and IFNα (n = 2). Source data are available for this figure.

**Figure S3.**
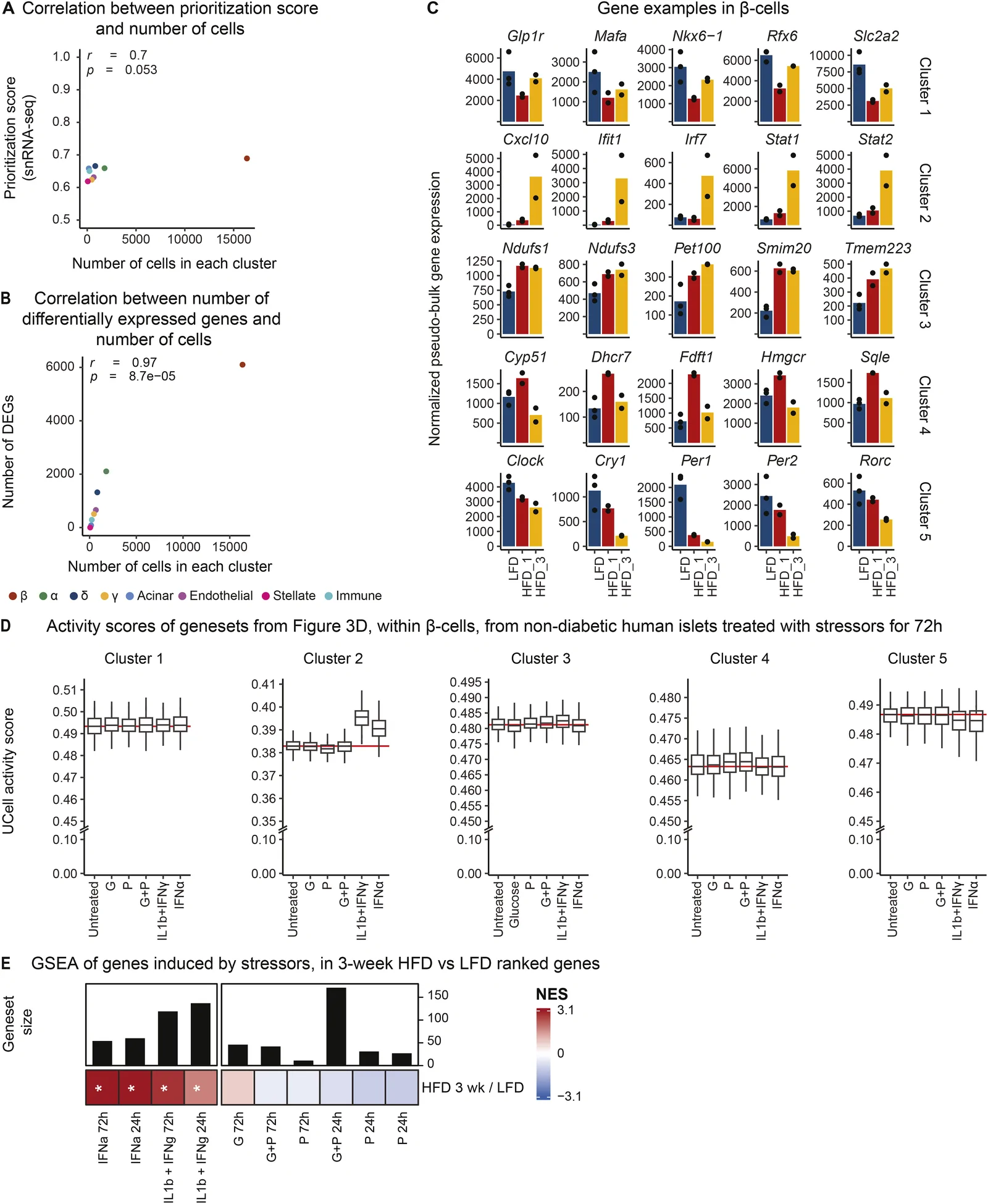
Related to Fig 3. **(A, B)** Scatter plots depicting the correlation between the number of cells within each cluster and the prioritization score calculated with Augur (A) or the number of differentially ex-pressed genes identified across conditions in each cell cluster (B). Pearson Correlation coefficient (r) and *P*-value (*P*) are written inside each plot and points are colored according to cell type as indicated on the figure. **(C)** Bar plot of examples of genes from the pathway analysis for each fuzzy cluster found in Fig 3D are shown. Gene expression is depicted for each replicate (points) using DESeq2 normalized pseudo-bulk counts across conditions. Bars indicate the mean expression. **(D)** Boxplot of the per cell gene activity score of gene clusters from Fig 3D within pancreatic β-cells from non-diabetic human donors which have been separated into groups treated with stressors; Glucose (G) (22 mM) and/or palmitate (P) (0.5 mM), interleukin-1beta (IL-1β) (1 ng/ml) and interferon gamma (IFNγ) (1,000 U/ml), or interferon alpha (IFNα) (2,000 U/ml) compared with untreated controls for 72 h (x-axis). Red lines indicate the median in the untreated samples. Number of donors in each condition at each time point: 72hr; Glucose (n = 2), palmitate (n = 2), glucose and palmitate = (n = 2), IL-1β and IFNγ (n = 2), IFNα (n = 2). **(E)** Heatmap showing the normalized enrichment score (NES) for the indicated gene sets among genes ranked by log fold change in expression between 3 wk of HFD and LFD. The bars indicate the size of the gene sets. *FDR ≤ 0.05.

To prioritize which cell types were most strongly affected by short-term HFD feeding, we used Augur (23), a machine learning-based tool, which prioritizes cell types based on their molecular responses. Augur identified β-cells as the most strongly perturbed cell type (Fig 3A). Since β-cells are the most abundant cell type in the dataset, we evaluated whether the prioritization scores calculated by Augur were confounded by cell type proportions and found no significant correlation between the number of cells in each cell type and the Augur prioritization score (Fig S3A). We also used differential expression analysis to prioritize cell types and identified the highest number of differentially expressed genes, totaling 6,101, between diet conditions in β-cells (Fig 3B). Although this method also prioritized β-cells as the most perturbed cell type, the result was strongly confounded by the number of cells in each cell type (Fig S3B). These results indicate that Augur is less biased than the number of differentially expressed genes for cell type prioritization in datasets with unbalanced cell type proportions, and that β-cells are most strongly affected by short-term HFD feeding.

Principal component analysis of pseudo-bulked β-cell transcriptomes indicates high reproducibility between biological replicates, and a clear separation between diet conditions along the first principal component, explaining 77% of the variance in the dataset (Fig 3C). Using fuzzy clustering, we partitioned the 6,101 differentially expressed genes in β-cells into five clusters, with distinct temporal profiles, and performed pathway analysis (Figs 3D and S3C). Cluster 1 consists of genes that are rapidly down-regulated in response to HFD, and these are associated with β-cell identity (e.g., *Nkx6-1 and Mafa*), glucose sensing (e.g., *Slc2a2* and *Irs2*) and insulin secretion (e.g., *Glp1r*). In contrast, genes in cluster 2 exhibit delayed upregulation and are associated with immune response signaling (e.g., *Stat1*, *Stat2*, *Irf7, Ifit1*, and *Cxcl10*). Specifically, the chemokine *Cxcl10* has been demonstrated to be secreted by islets from human organ donors with T2DM (26). Mechanistic studies have shown that *Cxcl10* is enriched in pro-inflammatory extracellular vesicles secreted by β-cells. These vesicles contribute to induction of pro-inflammatory gene expression in neighboring β-cells and enhanced recruitment of CD8^+^ T-cells and macrophages (27). Cluster 3 consists of genes that display sustained upregulation and is enriched for nuclear-encoded respiratory chain subunits of complex I (e.g., *Ndufs1* and *Ndufs3*), and genes involved in electron transport chain (ETC) complex IV assembly (e.g., *Smim20*, *Pet100*, and *Tmem223*). Genes in cluster 4 are transiently upregulated at 1 wk of HFD and are associated with sterol and cholesterol biosynthesis (*Hmgcr*, *Sqle*, *Dhcr7*, *Cyp51*, and *Fdtf1*). Lastly, cluster 5 represents genes that are continuously down-regulated, and include genes involved in transcriptional regulation of the circadian rhythm (e.g., *Clock*, *Per1/2*, *Cry1/2,* and *Rorc*). Circadian dysfunction has been implicated in the pathogenesis of T2DM, as islets from T2DM individuals show reduced expression of core clock genes such as *Per1* to *3* and *Cry1* (28). Collectively, these analyses indicate that islet β-cells undergo rapid and diverse transcriptional changes in response to HFD, including a rapid decrease in the expression of genes related to β-cell identity, glucose sensing, and circadian rhythm, as well as a rapid induction of genes involved in mitochondrial function, sterol biosynthesis and a slower induction of many inflammatory genes.

Given the growing recognition of the role of inflammatory cytokines and nutrient stressors in β-cell dysfunction, and T2DM risk (29 *Preprint*), we investigated their involvement in the transcriptional response to compensation as induced by short-term HFD. To this end, we used publicly available scRNA-seq data of islets of Langerhans from nondiabetic human donors (24, 25). These islets had been treated ex vivo with various stressors, including diverse metabolic and inflammatory stressors in parallel for 24 or 72 h. From each dataset, we extracted the β-cells and calculated gene module activity scores for each of the five clusters of DEGs (Figs 3D and S3D). Glucose (22 mM), palmitate (0.5 mM), or glucose and palmitate together did not induce gene programs similar to any of the HFD-induced clusters in murine islets in this study, suggesting that glucose and palmitate, when added ex vivo*,* cannot recapitulate the effects of short-term HFD. In contrast, treatment with the pro-inflammatory cytokines interferon alpha (IFNα) (2,000 U/ml), or the combination of interleukin 1beta (IL-1β) (1 ng/ml) and interferon gamma (IFNγ) (1,000 U/ml) induced a gene program in human β-cells that is similar to cluster 2 in our dataset. In support of this conclusion, gene set enrichment analysis (GSEA) of genes ranked by fold change in expression at 3 wk of HFD versus LFD showed specific enrichment of gene sets induced by pro-inflammatory cytokines (Fig S3E). Collectively, this indicates that short-term HFD induces a rapid pro-inflammatory response in β-cells.

### Pro-inflammatory eRegulons are activated in β-cells following short-term high-fat diet

**Figure 4.**
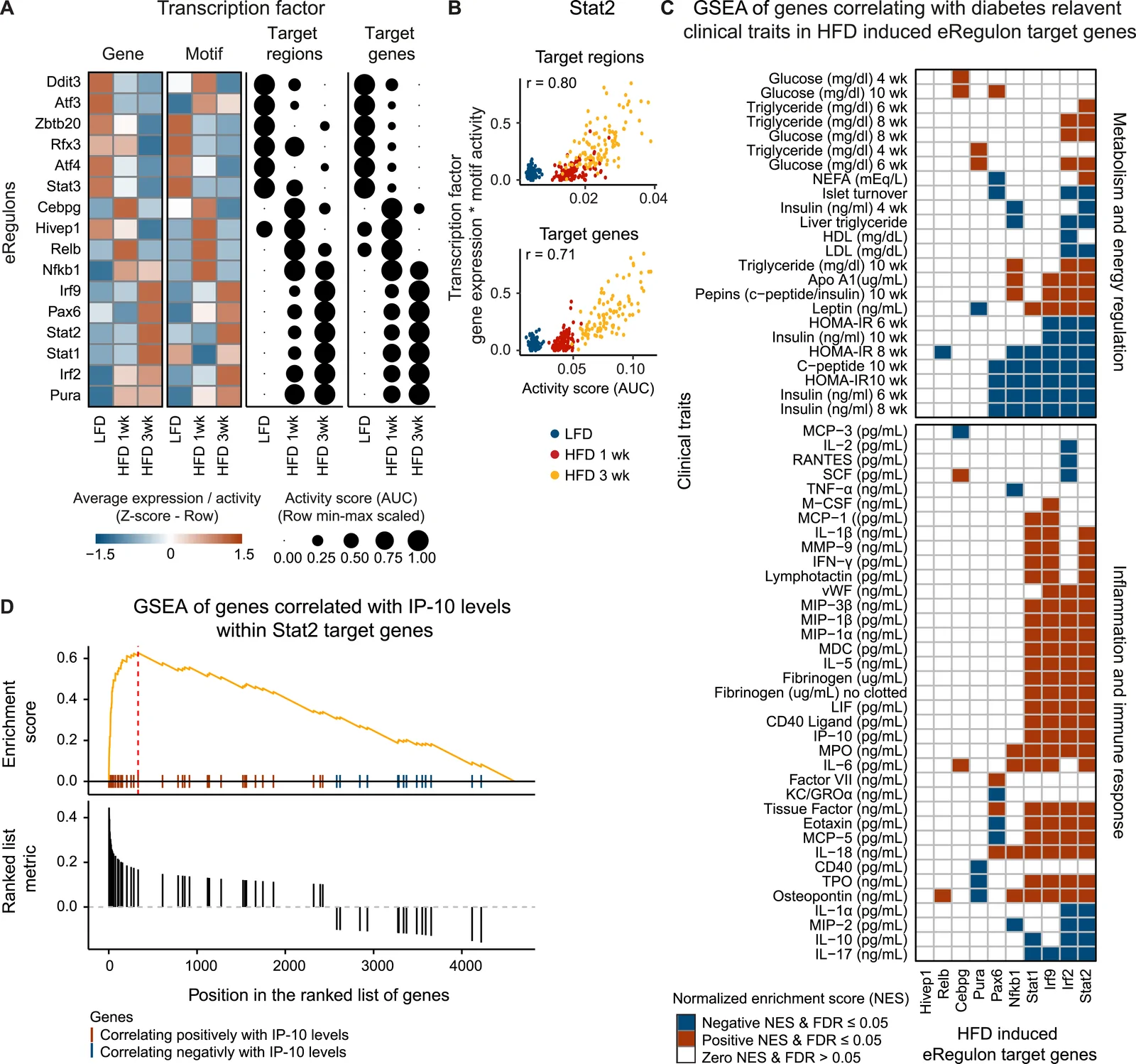
Immune and inflammatory transcription factors drive transcriptional changes in β-cells in response to short-term high-fat diet. **(A)** Left panel: Heatmap displaying z-transformed normalized average gene expression (left) and motif activity (right) of eRegulon transcription factors across conditions. Right panel: Dot plot illustrating row min–max scaled average activity score (AUC) of target enhancers (left) and target genes (right), representing the gene set activity for each eRegulon per condition. Dot size corresponds to AUC. **(B)** Pearson correlation analysis of semi-pseudo-bulked data (100 meta-cells per condition, each with 10 cells), from the Stat2 eRegulon. The y-axis depicts the min–max scaled product of semi-pseudo-bulked transcription factor, Stat2, gene expression, and motif activity. The x-axis depicts the activity score for Stat2 target enhancers (top) and Stat2 target genes (bottom). Points are colored according to condition (LFD: blue, HFD 1 wk: red, and HFD 3 wk: yellow). **(C)** Heatmap illustrating gene set enrichment analysis (GSEA) of genes that correlate positively (correlation ≥0.1) or negatively (correlation ≤−0.1) with clinical traits of diabetes from the Attie lab diabetes database (http://diabetes.wisc.edu/correl_f2.php) within high-fat-diet (HFD) induced eRegulon target genes. Genes associated with metabolism and energy regulation (top panel) as well as inflammation and immune response (bottom panel) are shown. Gene sets that are significantly enriched (false discovery rate [FDR] ≤ 0.05) within an eRegulon gene set are colored (blue if NES >0 otherwise red). Rows and columns were clustered using hierarchical clustering, and rows were split by functional category (metabolism/energy regulation vs. inflammation/immune response). Dendrograms are not displayed. **(D)** GSEA enrichment plot of the Interferon γ-induced protein 10 (IP-10, also known as CXCL10) gene set enriched within Stat2 target genes. Top: The y-axis represents the enrichment score, while the x-axis shows a rug plot of genes from the IP-10 gene set, found within the Stat2 target genes, ranked by their correlation with IP-10 levels, from high positive to low negative, and are colored based on their correlation score: positive (red) or negative (blue). The orange curve represents the running sum of the enrichment score across the ranked genes. The peak of this curve (indicated by the red dotted line) represents the maximum enrichment score for the gene set. Bottom: A bar plot illustrating the ranking of genes based on their correlation with IP-10 levels. Source data are available for this figure.

**Figure S4.**
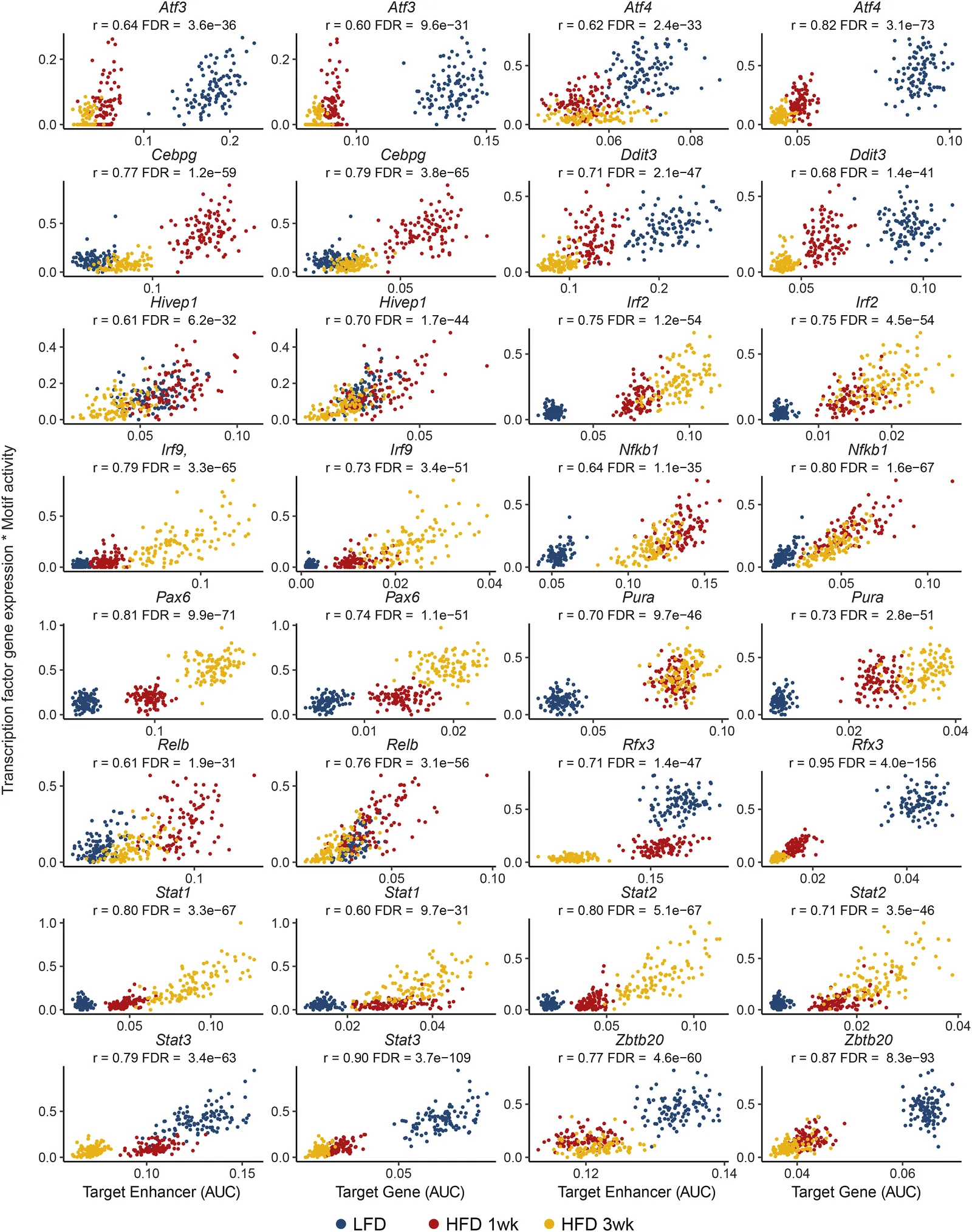
Related to Fig 4. Correlation analysis using semi-pseudo-bulked data (100 meta-cells per condition, each with 10 cells). The correlation analysis was performed using the min–max scaled product of semi-pseudo-bulked transcription factor gene expression and of transcription factor motif activity with activity scores (AUC) for target genes and enhancers from SCENIC+ (computed using AUCell) for each eRegulon (y-axis). Nuclei are colored according to conditions as indicated on the figure. Pearson Correlation coefficient (r) is written inside each plot.

To identify transcriptional regulators of the rapid HFD-induced response in β-cells, we applied SCENIC+ (30), which infers gene-regulatory networks by identifying enhancer-driven regulons (eRegulons) containing transcription factors and their associated target enhancers and genes. We applied this approach to our paired snRNA-seq and snATAC-seq data and identified a total of 43 eRegulons. Filtering of these resulted in 16 high-quality eRegulons, all of which were classified as activating eRegulons by SCENIC+ (Fig 4A and Table S3). High quality was determined by how well the expression of a transcription factor, and its motif activity, correlated with its activity scores from its potential target enhancers and genes across the different conditions (Figs 4B and S4). Therefore, high-quality eRegulons are those where the transcription factors are active and expressed in conditions where their target genes are also expressed, and the target regions are accessible.

Table S3. Summary of eRegulons.

Following HFD feeding, several transcription factors, which have been implicated in β-cell function, display decreased gene expression and motif activity along with decreased target enhancer and target gene activity. These include Regulatory Factor X3 (*Rfx3*), which has been reported to play a role in islet development, and mature β-cell function, including the regulation of glucokinase gene expression (31).

In contrast to the down-regulated eRegulons, the transcription factors driving upregulated eRegulons are not core regulators of β-cell identity and function. Instead, they are pro-inflammatory transcription factors, such as the NF-κB family (*Nfkb1* and *Relb*), which display a transient increase in expression, motif activity, and target enhancer and gene activity scores, as well as mediators of type I interferon (IFN) signaling, such as *Irf2*, *Irf9*, *Stat1*, and *Stat2*, which exhibit a delayed response to HFD, with some increase in target enhancer activity and target gene activity after 1 wk of HFD. Type I IFN signaling is classically involved in innate antiviral responses but has also been implicated in the early stages of type 1 diabetes mellitus (T1DM) autoimmunity (32, 33), as well as in the dysregulation of insulin sensitivity and energy metabolism in diet-induced obesity in mice (34, 35).

We next explored whether the HFD-induced eRegulons are associated with diabetes-related clinical traits. To do this, we made use of the Attie lab diabetes database (36), which includes bulk RNA-seq data of islets of Langerhans from obese B6:BTBR F2 mice, that either positively or negatively correlate with diabetes-related clinical traits. Genes with a Pearson correlation coefficient greater than 0.1 (positive correlation) or less than −0.1 (negative correlation) were included in the gene lists (36). This means that a gene set that correlate positively with blood glucose levels indicates that increased expression of these genes is associated with higher blood glucose levels. Conversely, a negative correlation means that increased expression of these genes is associated with lower blood glucose levels or decreased expression of these genes is associated with high blood glucose levels. GSEA revealed that the target genes of HFD-induced eRegulons are enriched in genes that either positively or negatively correlate with diabetes-related clinical traits. The analysis showed that the *Irf2*, *Irf9*, *Stat1,* and *Stat2* eRegulons were enriched for gene sets that correlate negatively with clinical traits related to insulin secretion, such as serum insulin and C-peptide levels, and *Irf2* and *Stat2* eRegulons are enriched for gene sets that correlate positively with plasma levels of glucose, non-esterified fatty acids (NEFA), and triglycerides. Furthermore, *Irf2*, *Irf9*, *Stat1*, and *Stat2* are also enriched for gene sets that correlate positively with circulating levels of chemokines, cytokines, and pro-inflammatory proteins, including for example interferon gamma-induced protein 10 (IP-10, *Cxcl10*), IL-1β, interleukin-6 (IL-6), Osteopontin, and IFNγ (Fig 4C and D). These observations are concordant with the established role of these transcription factors in inflammatory signaling pathways (37, 38, 39), and suggest a causal link between systemic pro-inflammatory molecules and regulation of β-cell function.

### β-cells display a heterogenous response to inflammation

**Figure 5.**
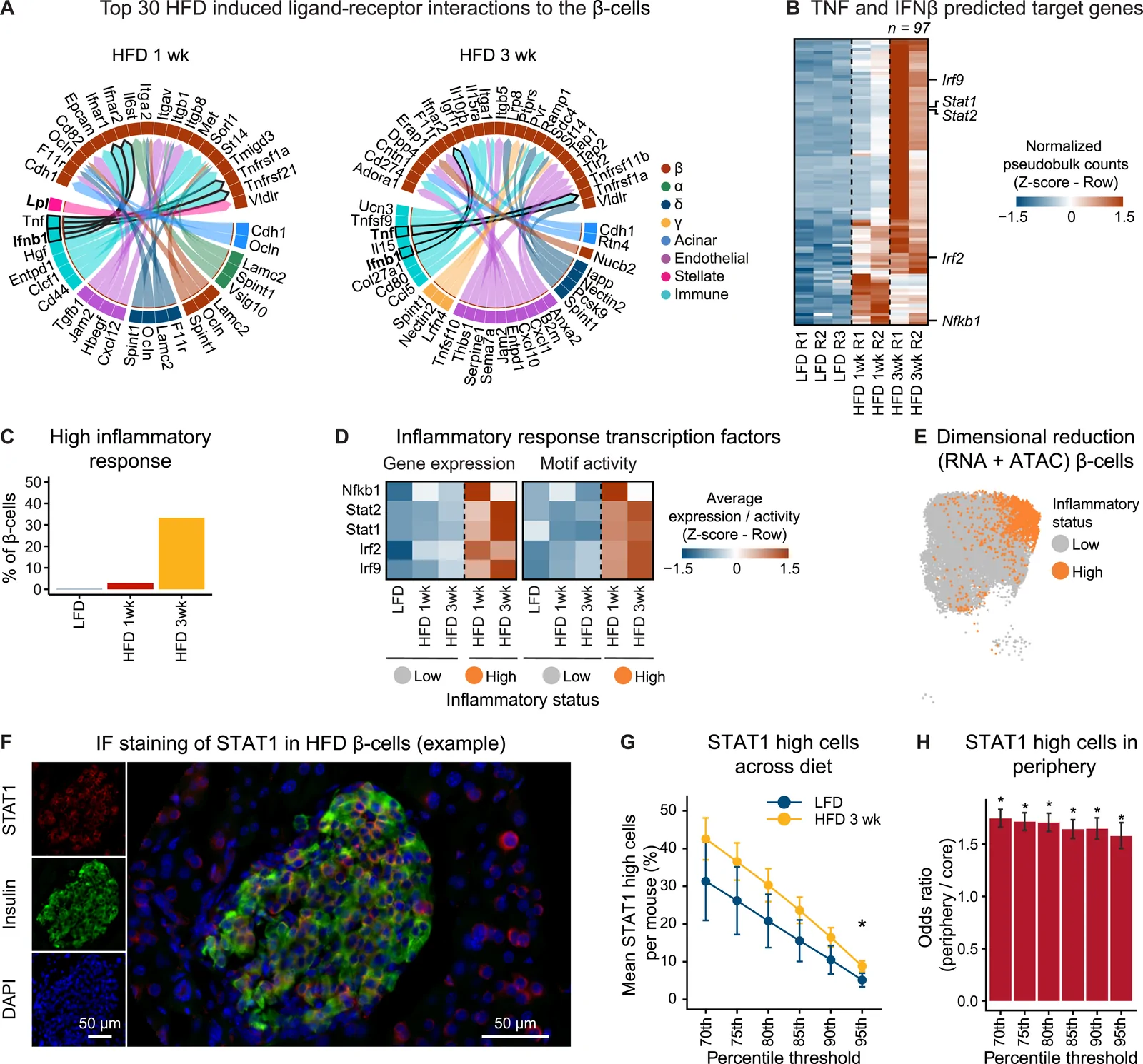
Identification of β-Cell subclusters based on inflammatory response. **(A)** Circos plot of the top 30 predicted interaction links from all cell types to the β-cells from one week (1 wk) or three weeks (3 wk) of high-fat diet (HFD). **(B)** Heatmap displaying z-transformed normalized pseudo-bulk gene counts for putative tumor necrosis factor (Tnf) and interferon beta (ifnb1) target genes. **(C)** Percentage of high inflammatory response β-cells at each condition. **(D)** Heatmap displaying Z-transformed average gene expression (left) and motif activity (right) of inflammatory-related eRegulon transcription factors across conditions and β-cell low (gray dot) or high (orange dot) inflammatory subpopulation. **(E)** Uniform manifold approximation and projection (UMAP) visualization of β-cells colored by inflammatory status. **(F)** 20× immunofluorescence (IF) image of pancreas sections stained for STAT1 (red), insulin (green), and DAPI (blue). Individual channels on the left and a merge of all channels on the right. **(G)** Average percentage of STAT1-high β-cells per mouse across percentile thresholds (70th–95th) in LFD- and HFD-fed mice (3 wk; n = 4/diet). STAT1-high was defined as nuclear or perinuclear STAT1 mean pixel intensity exceeding the given threshold. Error bars indicate mean ± standard deviation (SD). Asterisks indicate significant diet differences at each threshold (Wilcoxon test, *P* < 0.05). **(H)** Odds ratio of a β-cell being STAT1-high in the periphery versus the core of HFD islets containing both populations, from mixed-effects logistic regression fit per percentile threshold. Error bars show 95% confidence interval. Significance was assessed by permutation test (999 permutations, shuffling STAT1 status within islet); asterisks denote two-tailed empirical *P* < 0.05. n = 488–348 islets from 4 HFD mice. Source data are available for this figure.

To identify potential intrinsic sources of pro-inflammatory signals in the islets, we used NicheNet (40 *Preprint*) to prioritize ligand-receptor pairs that most likely account for differential gene expression in β-cells between LFD versus 1 or 3 wk HFD, and to predict their downstream target genes. NicheNet identified tumor necrosis factor (TNF) and interferon beta (IFNβ) as immune cell-derived ligands within the top 30 prioritized interactions after both 1 and 3 wk of HFD feeding (Fig 5A). The 97 predicted target genes of TNF and IFNβ are all induced by HFD, and the majority are most highly expressed after 3 wk of HFD feeding (Fig 5B). Among the target genes are the pro-inflammatory transcription factors themselves, which suggests that there is a positive feedback loop that strengthens the inflammatory response.

**Figure S5.**
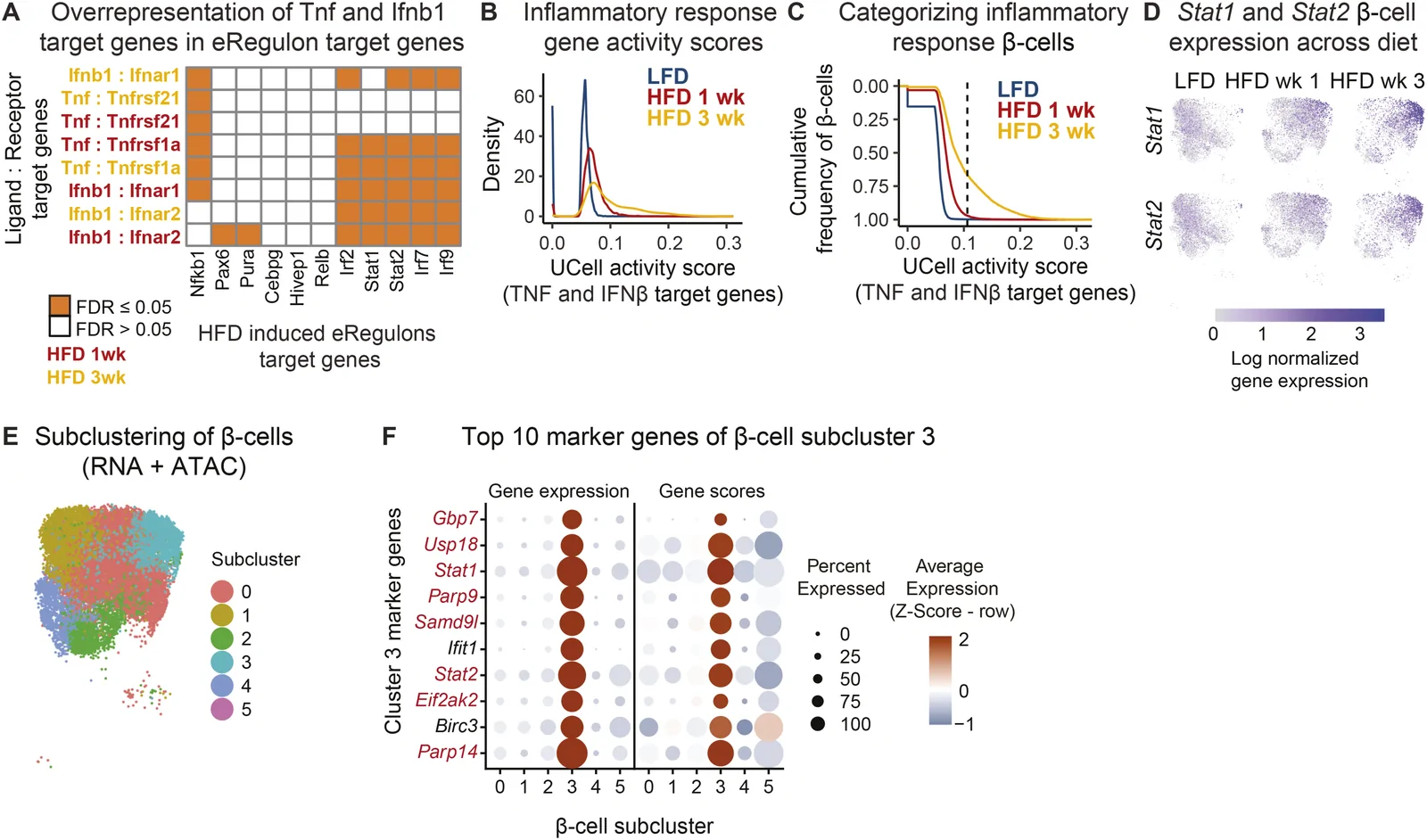
Related to Fig 5. **(A)** Overrepresentation analysis of target genes from the top TNF and IFN-β interaction partners predicted at 1-wk HFD (red, HFD 1 wk) and 3 wk HFD (yellow, HFD 3 wk) and eRegulon target genes. Significance was determined using Fisher’s exact test (FDR < 0.05). **(B)** Density plot showing the distribution of gene activity scores of inflammatory response genes (TNF and IFNβ predicted target genes) split into each condition. **(C)** Empirical cumulative distribution function plot of gene activity scores of inflammatory response genes (TNF and IFNβ predicted target genes) split into each condition. The vertical line indicates the threshold used to define cells with high inflammatory signature. **(D)** UMAP embedding of β-cells colored by normalized expression of Stat1 and Stat2, split by conditions. Color scale is shared across all panels for direct comparison. Points are ordered by expression so that high-expressing cells are plotted on top. **(E)** UMAP visualization of β-cell subclusters. **(F)** Dot plot showing the percentage of β-cell subcluster cells expressing the gene (dot size) and scaled average gene expression (dot color) of the top 10 marker genes for cluster 3 in the left panel, as well as the average gene score, calculated based on the chromatin accessibility of regulatory elements in the vicinity of the gene, of top 10 markers in the right panel. Genes colored in red represent genes that are a part of TNF and IFNβ target genes.

Using overrepresentation analysis, we compared activated predicted target genes of TNF and IFNβ ligand-receptor interactions with the target genes of HFD-induced eRegulons. We found the predicted TNF and IFNβ target genes to be significantly enriched (false discovery rate (FDR) ≤ 0.05) for genes predicted to be target genes of the inflammatory eRegulons driven by *Nfkb1*, *Irf2*, *Irf9*, *Stat1*, and *Stat2*, but not *Relb* (Fig S5A). These results indicate that the transcriptional effects of TNF and interferon signaling are mediated by the activities of these transcription factors to modulate inflammatory gene expression patterns in response to short-term HFD.

There are conceptually two distinct mechanisms that can lead to an increased inflammatory response; either all cells have a modest response, or a subset of cells have a strong response. To explore which of these mechanisms are driving the inflammatory response in β-cells during short-term HFD feeding, we calculated a gene module activity score in every β-cell, using the TNF and IFNβ target genes. Generally, all β-cells in the HFD groups show slightly elevated scores, indicating that HFD influences the overall inflammatory response in β-cells across conditions. However, after 3 wk of HFD, the distribution of these scores was right-skewed, suggesting that a subset of β-cells exhibit a pronounced inflammatory response at this time point (Fig S5B). By sorting the gene module activity scores for the inflammatory response gene set from all β-cells across HFD conditions, we determined the knee point of the curve as a threshold (gene module activity score ≥0.106) for segregating the β-cells into two subpopulations with high or low inflammatory response (Fig S5C). This analysis shows that there is an increase in the percentage of β-cells belonging to the high inflammatory subpopulation during exposure to HFD, from ∼0% under LFD conditions to ∼3% following 1 wk of HFD, and ∼33% following 3 wk of HFD (Fig 5C). Consistently, both the average gene expression and the inferred motif activity of the transcription factors *Nfkb1*, *Irf2*, *Irf9*, *Stat1*, and *Stat2* are higher in the high inflammatory subpopulation compared with the low inflammatory subpopulation, which was not different from the LFD condition (Fig 5D). High inflammatory β-cells occupy a distinct region on the UMAP embedding (Fig 5E), which is also enriched for expression of *Stat1* and *Stat2* (Fig S5D). To assess whether this inflammatory subtype emerged independently of the gene signature used for its definition, we performed unsupervised clustering on the β-cell subset. Louvain clustering (resolution 0.2) identified six distinct populations, with cluster 3 enriched for high inflammatory cells (60.1% versus 0.6–9.1% in other clusters (Fig S5E). Of the 58 upregulated genes in cluster 3, 18 were TNF and IFNβ target genes, with 8 among the top 10 marker genes ranked by percentage expressed, FDR, and log2 fold-change (Fig 5SF).

The presence of high inflammatory response β-cells was validated in vivo using high-throughput immunofluorescence microscopy of pancreatic tissue from mice fed LFD or HFD for 3 wk (n = 4 per diet) (Table S4). Tissues were stained for insulin (β-cell and islet marker), STAT1 (inflammatory response marker), and DAPI (nuclei) (Fig 5F). On average, we identified 210.5 islets (range: 64–149) and 1,588.25 cells (range: 6,606–15,670) per mouse. In insulin-positive cells, we quantified the mean pixel intensity (MPI) of STAT1 within the nucleus (defined using DAPI) and the perinuclear cytoplasmic region, as STAT1 is localized in both the nucleus and the cytoplasm. Across a wide range of thresholds, HFD-fed mice had a higher percentage of STAT1 high β-cells compared with LFD controls, reaching statistical significance at most stringent threshold (*P* ≤ 0.05) (Fig 5G). To characterize the spatial distribution of inflammatory response β-cells within islets, we tested whether STAT1 high cells were preferentially localized at the islet periphery or the core. Cells were classified as either periphery or core cells based on their distance to the islet boundary, with the 30% closest cells annotated as cells in the periphery and the remaining cells as core cells. Using a binomial mixed model accounting for variation between islets, we investigated whether the odds of a cell being STAT1 high in the periphery relative to the core. We found that periphery cells had significantly higher odds of being STAT1-high than core cells (*P* ≤ 0.05) (Fig 5H).

Table S4. Summary of mice used for immunofluorescence microscopy.

### Human β-cells display a heterogeneous inflammatory signature in diabetes

**Figure 6.**
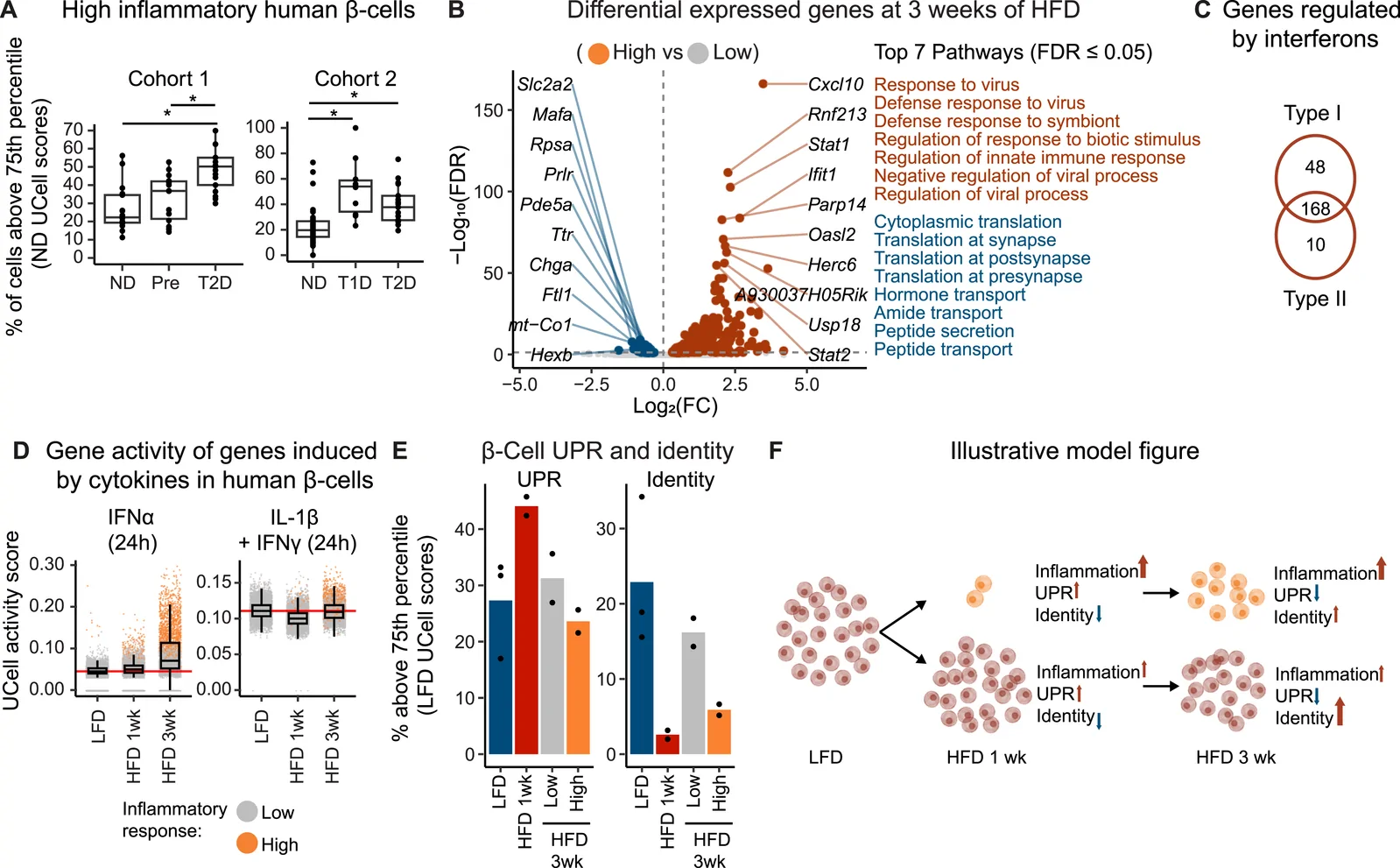
Characterization of the inflammatory β-cell subpopulation and its relationship to β-cell identity and human diabetes. **(A)** Boxplot of the prevalence of β-cells with high TNF and IFNβ target gene activity, defined as gene activity scores above the 75th percentile from non-diabetic β-cells stratified by donor and disease status. Cohort 1 (41 *Preprint*): Non-diabetics (ND, n = 17). Prediabetics (Pre, n = 14). Type 2 diabetics (T2D, n = 17). Cohort 2 (42): Non-diabetics (ND, n = 38). Type 1 diabetics (T1D, n = 10). Type 2 diabetics (T2D, n = 17). Each point represents a donor. **(B)** Left panel: Volcano plot depicting differentially expressed genes (DEGs) in high (orange dot) versus low (gray dot) inflammatory response β-cell subpopulation at 3-wk HFD (HFD 3 wk). The horizontal line indicates false discovery rate (FDR) = 0.05 and the vertical line indicates log_2_ fold change (FC) = 0. Top 10 upregulated (red) and down-regulated (blue) genes are annotated. Right panel: The top seven most significant (FDR ≤ 0.05) pathways from Gene Ontology biological processes for upregulated (red) and down-regulated (blue) genes. **(C)** Venn diagram showing results of enrichment search in INTERFEROME (43) identifying an overlap between upregulated genes in high inflammatory β-cell subpopulation at 3 wk of HFD, and genes known to be involved in interferon signaling pathways (type I, and II). **(D)** Boxplot of the per β-cell gene activity score per condition using sets of genes that are significantly upregulated in pancreatic β-cells from non-diabetic human donors which have been treated with stressors; Interleukin-1beta (IL-1β) (1 ng/ml) and Interferon gamma (IFNγ) (1,000 U/ml), Interferon alpha (IFNα) (2,000 U/Ll) compared with untreated controls for 24 h (24, 25). Gray dots represent β-cells in the low inflammatory subpopulation and orange dots represent β-cells in the high inflammatory subpopulation. Red lines indicate the median activity score in the LFD samples. **(E)** Bar plot showing the mean prevalence of β-cells with high unfolded protein response (UPR) (left) or β-cell identity (right) as defined by the percentage (%) of cells above the 75th percentile of gene activity scores, using genes related to UPR or identity, within the low fat diet (LFD) β-cells, for each condition: LFD, HFD 1 wk and HFD 3 wk, where HFD 3 samples are subdivided by inflammatory state; low-inflammatory (low) and high-inflammatory (igh). Each point represents a replicate. **(F)** Illustration of proposed mechanism: Following 1 wk of HFD (HFD 1 wk) feeding UPR-mediated stress and transcriptional down-regulation of β-cell identity genes in most β-cells, as well as induction of an inflammatory response in a small subset of β-cells (orange cells). After 3 wk of HFD (HFD 3 wk), the UPR stress is resolved, but the subset of β-cells with an inflammatory response is increased. In β-cells without an inflammatory response, β-cell identity gene transcription is partially restored, but not in β-cells with an inflammatory response. Asterisk indicates FDR-adjusted *P*-value ≤0.05. Source data are available for this figure.

**Figure S6.**
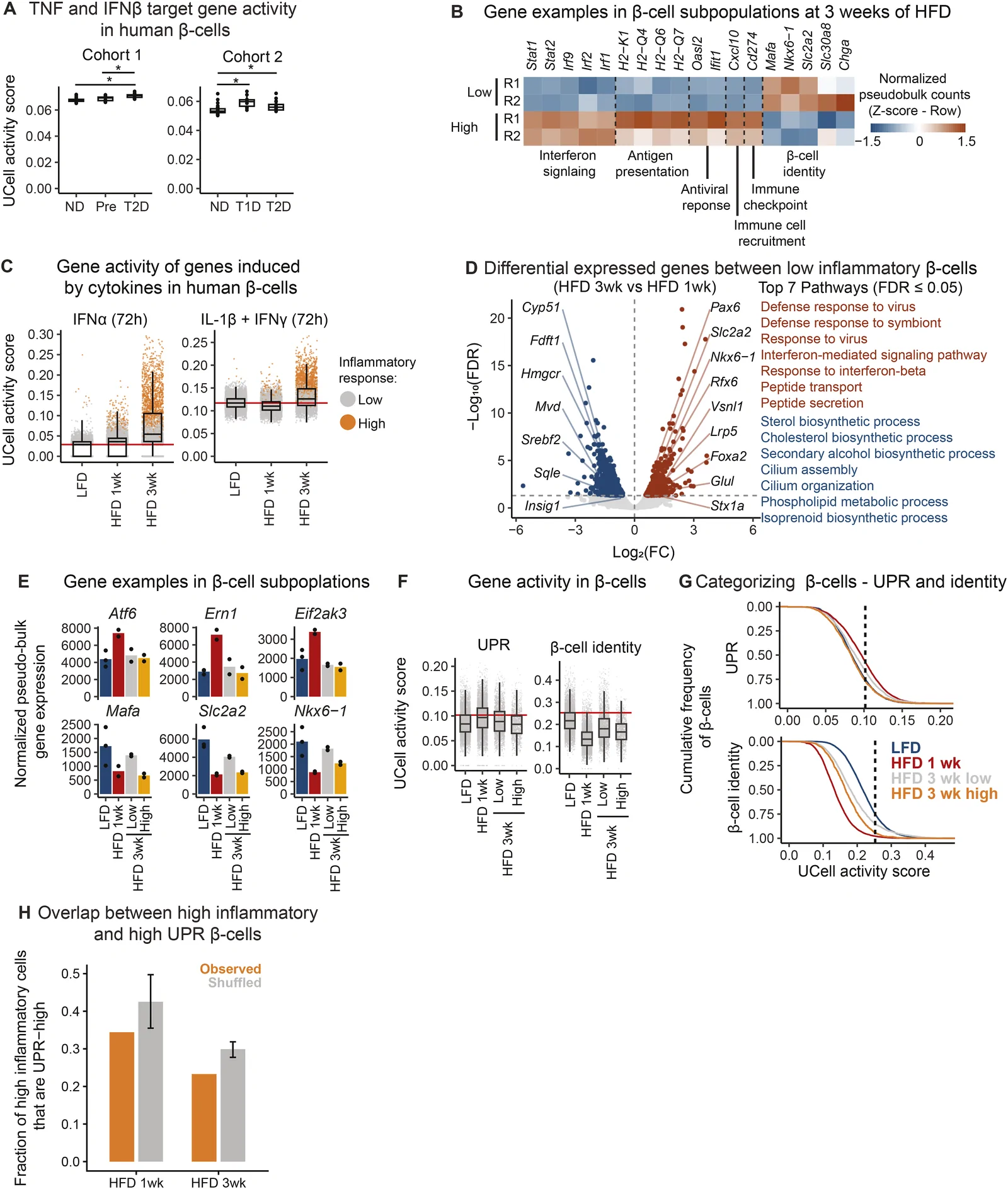
Related to Fig 6. **(A)** Boxplot of mean gene activity scores using genes for TNF and IFNβ target genes in β-cells from human donors, stratified by donor and disease status in the same way as in Fig 5E. **(B)** Heatmap displaying row-scaled z-scores of DESeq2 normalized pseudo-bulk counts for gene examples significantly changed between HFD 3 wk β-cells with low or high inflammatory signature. **(C)** Boxplot of the per β-cell gene activity score per condition, using sets of genes that are significantly upregulated in pancreatic β-cells from non-diabetic human donors which have been treated with stressors; IL-1β (1 ng/ml) and IFNγ (1,000 U/ml), IFNα (2,000 U/ml) compared with untreated controls as in Fig 6D. Red lines indicate the median in the untreated LFD samples. **(D)** Volcano plot depicting differentially expressed genes between β-cell with a low inflammatory response subpopulation at 3 and 1 wk (3 vs. 1 wk). The horizontal line indicates false discovery rate (FDR) = 0.05 and the vertical line indicates log2 fold change (FC) = 0. Selected down-regulated (red) and down-regulated (blue) genes are annotated. Right panel: The top 10 most significant (FDR < 0.05) pathways from Gene Ontology biological processes for upregulated and down-regulated genes. **(E)** Bar plot of examples of genes related to UPR and β-cell identity. Gene expression is depicted for each replicate (points) using normalized pseudo-bulk counts across conditions, where HFD 3 wk is further divided into low inflammatory (low) and high inflammatory (high) subpopulations. **(F)** Boxplot of gene activity scores using genes related to either UPR (left) or β-cell identity (right), for each condition LFD, HFD 1 wk and HFD 3 wk, where HFD 3 wk samples are subdivided by inflammatory state; low inflammatory (low) and high-inflammatory (high). Red line indicates the 75th percentile of scores from LFD. Each point represents a nucleus. **(G)** Empirical cumulative distribution function plot of gene activity scores using genes related to either UPR (top) or β-cell identity (bottom) split into each condition. The vertical line indicates the threshold used to define cells with high UPR or identity signature. **(H)** Bar plot of the fraction of high inflammatory β-cells that are also classified as high UPR, for HFD 1 wk and HFD 3 wk. The observed fraction (orange) is shown together the fraction expected if the two classifications were unrelated (gray), obtained by randomly reassigning the UPR labels among β-cells within each condition 1,000 times. Gray bars show the mean of the 1,000 permutations and error bars indicate the interval containing the middle 95% of permuted values. Asterisk indicates FDR-adjusted *P*-value ≤0.05.

To explore whether the inflammatory response observed in mouse islets in response to HFD is also observed in human β-cells, we used scRNA-seq data from two previously published studies. The first study, Cohort 1, compares islets from individuals non-diabetic subjects, prediabetes T2DM, or T2DM (41 *Preprint*), whereas the second study, Cohort 2, compares islets from individuals with or without T1DM or T2DM (42). We used the TNF and IFNβ target genes to calculate the gene module activity score in every β-cell and used the 75th percentile gene module activity score in non-diabetic β-cell as a threshold for defining cells with high inflammatory response (Figs 6A and S6A). In cohort 1, there was a tendency for an increased prevalence of high inflammatory β-cells in prediabetic compared with non-diabetic β-cells, though it was not statistically significant. In T2DM subjects, the increase was significant (*P* < 0.05). In cohort 2, the prevalence of high inflammatory β-cells was also significantly increased (*P* < 0.05) in both T2DM and T1DM β-cells, however to a much larger degree in the T1DM β-cells (Figs 6A and S6A). Collectively, this indicates that a subset of β-cells with a high inflammatory signature exists in humans and is related to the development of diabetes.

To explore the differences between high and low inflammatory response β-cells, we performed differential gene expression analysis between these two subpopulations at 3 wk of HFD and found that 265 genes were significantly upregulated in the high inflammatory subpopulation, while 63 genes were down-regulated (Fig 6B). As expected, the genes upregulated in high relative to low inflammatory response β-cells are key regulators of interferon signaling, as well as genes related to antigen presentation, antiviral responses, immune cell recruitment, and immune checkpoint regulators (Fig S6B). Of the 265 upregulated genes, 226 were described in the INTERFEROME database (43) as genes whose expression is induced by treatment with interferons (168 by both type I and II IFNs, 48 by type I, and 10 by type II) (Fig 6C). To further support that this subset of β-cells is activated by type I IFN signaling, we used public data (24, 25) to define genes activated in human β-cells after acute type I IFN signaling (using IFNα) or after acute type II IFN signaling (using IL-1β and IFNγ). We observed a strong increase in gene module activity scores in high inflammatory response β-cells for type I IFN signaling but not type II IFN signaling (Figs 6D and S6C).

The 63 down-regulated genes, whose expression was induced in low inflammatory β-cells, were associated with β-cell identity (e.g., *Mafa* and *Nkx6-1*), glucose sensing (e.g., *Slc2a2*), and insulin secretion (e.g., *Slc30a8* and *Chga*) (Fig S6B). Genes associated with β-cell identity and function was also expressed at higher levels in low inflammatory response β-cells at 3 wk relative to 1 wk of HFD (Fig S6D), thereby supporting previous studies indicating a transient reduction in the expression of these genes in islets after 1 wk of HFD, followed by restoration at 2 and 3 wk (14). When insulin demand increases, β-cells may reach ER protein folding capacity, leading to misfolded proinsulin accumulation. To manage this stress, β-cells have been reported to activate an unfolded protein response (UPR) (6, 44). Comparison of gene expression in low inflammatory β-cells from LFD controls and 1 wk of HFD revealed increased expression of the three key components of the UPR: *Atf6* (ATF6), *Ern1* (IRE1), and *Eif2ak3* (PERK) (45) (Fig S6E). We identified a gene set for ATF6, IRE1, and PERK-mediated UPR (Table S5), and used the 75th percentile gene module activity score in LFD β-cell as a threshold for defining cells with high UPR. The prevalence of β-cells with high UPR was transiently increased at 1 wk of HFD, which returned to levels comparable to the LFD control after 3 wk of HFD regardless of inflammatory state (Figs 6E and S6F and G). High UPR β-cells overlap less than expected by random with high inflammatory response β-cells suggesting that these are distinct transcriptional responses to distinct stressors (Fig S6H). Using the same approach to define β-cells with high expression of key β-cell identity genes (Table S5), we observed a transient reduction in the prevalence of β-cells with high identity after 1 wk of HFD. However, as with the UPR response, this reduction was reversed by 3 wk of HFD in low-inflammatory β-cells, with prevalence returning to LFD levels. In contrast, no such resolution was observed in high-inflammatory β-cells, where the prevalence was slightly elevated compared with 1 wk of HFD.

Table S5. Unfolded protein response and beta-cell identity genesets.

Collectively, these results point to a mechanism where 1 wk of HFD feeding increase insulin secretion from β-cells, which is accompanied by UPR-mediated stress and transcriptional down-regulation of β-cell identity genes in most β-cells, as well as induction of an inflammatory response in a small subset of β-cells. After 3 wk of HFD, the UPR stress is resolved, but the subset of β-cells with an inflammatory response is increased. In β-cells without an inflammatory response, β-cell identity gene transcription is partially restored, but not in β-cells with an inflammatory response (Fig 6F).

## Discussion

One of the hallmarks of the development of T2DM in humans is the transition from functional compensation to decompensation in pancreatic β-cells. In this study, we aim to identify the signals and regulators of this transition in a murine model of diet-induced obesity and insulin resistance by studying the effects of short-term HFD. At the physiological level, we observed that HFD had a rapid and significant impact on metabolic homeostasis consistent with previous research (14, 15). After 1 wk of HFD, we observed hyperglycemia and impaired glucose tolerance relative to control mice as measured by IPGTT, indicative of insulin resistance. After 3 wk of HFD, we observed a slight reduction in the secretory capacity of β-cells relative to 1 wk of HFD, as measured by SPINA-Gβ, together with a modest reduction in fasting serum insulin levels. These observations suggest that, despite continued metabolic stress, the compensatory response may begin to wane over time. This functional compensation occurs in conjunction with strong induction of transcriptional programs related to UPR, cholesterol biosynthesis and oxidative phosphorylation, as well as repression of β-cell identity and function genes. After 3 wk of HFD, we observed a reduction in the secretory capacity of β-cells relative to the controls as measured by SPINA-Gβ and a slight reduction in fasted serum insulin levels. These observations on the phenotypic level underscore that our model induces functional compensation, which transitions into early decompensation. To identify the signals and regulators of these functional transitions in islets of Langerhans, we mapped the transcriptome and the regulatory epigenome (measured through chromatin accessibility) using single nucleus multiomics.

Of all cell types in the islet of Langerhans, the β-cells undergo the largest transcriptional changes highlighting that β-cells play a pivotal role during functional transitions. Several recent studies have similarly reported decreased expression of genes associated with β-cell identity and function in both rodent and human β-cells in response to different metabolic stressors both in vivo and in vitro (46, 47, 48, 49, 50). This has been suggested to be linked to the activation of UPR (51, 52), which may repress β-cell identity and function genes either through activation of transcriptional repressors, or through a mechanism known as squelching, where cofactors upon strong transcriptional activation of a novel gene set are redistributed from cell-type specific enhancers leading to “passive repression” (53). Using our data, we show that activation of UPR (and other biological processes, including cholesterol biosynthesis, and oxidative phosphorylation) and repression of identity and function genes in β-cells occurs during the initial functional compensation phase in vivo. Thus, our observations support the hypothesis that β-cells secreting a large amount of insulin, simultaneously undergo transcriptional repression of identity and function genes mediated by UPR activation, oxidative and ER stresses through accumulation of misfolded proinsulin, and generation of reactive oxygen species due to high metabolic flux (14, 44, 45, 54).

After 3 wk of HFD, we observed a decompensatory response as evidenced by a reduction in the secretory capacity of β-cells relative to the controls measured by SPINA-Gβ and a slight reduction in fasted serum insulin levels. Since these mice still displayed a relative reduction in glucose tolerance and hyperglycemia, indicative of insulin resistance, this reduction in β-cell function may represent β-cell exhaustion. This decompensation occurs in conjunction with the resolution of transcriptional induction of UPR.

Previous research suggests that the resolution of ER stress and UPR leads to normalization of transcription of β-cell identity and function genes (51). In our data, we observed a dichotomous response, where approximately two-thirds of the β-cells restore the expression of identity genes to a large degree, whereas in the remaining β-cells, restoration of expression of identity genes is significantly blunted. The β-cells with blunted restored expression of identity genes upon UPR resolution are characterized by a strong pro-inflammatory response, which we map to IFN-α signaling.

Collectively, our observations point to a two-hit mechanism. In the early phase of insulin resistance, the increased demand for insulin is compensated by β-cells through increased insulin secretion, which simultaneously leads to transcriptional repression of β-cell identity and function genes potentially through squelching due to UPR activation and other gene programs. Upon β-cell exhaustion, the induced programs, including UPR, are resolved, but in a large subset of β-cells, a strong inflammatory response takes over and maintains the β-cell in a squelched state, thereby preventing normalization of expression of β-cell identity and function genes.

It has been established that obesity and T2DM in humans is associated with chronic low-grade inflammation, characterized by increased circulating levels of cytokines and chemokines (55, 56, 57), along with increased activation and recruitment of pro-inflammatory immune cells to multiple organs, including the endocrine pancreas (58, 59, 60). Studies in mice have reported increased levels of circulating cytokines following just three days of HFD (61) and increased macrophage infiltration in murine islets after 1 wk of HFD (62) as well as in human islets from individuals with T2DM (59).

In our data, we did not observe an increased number of immune cells in islets upon HFD, but caution is warranted due to the limitations of single-nucleus techniques in identifying rare cell types. For rare cell populations, the detection rate may not accurately reflect true proportions due to limited sampling or small variations during library preparation. However, based on inference of eRegulons, cell-cell communication analyses and association between metabolic traits and gene expression, we identified type I IFN signaling as the primary inflammatory signal in a subset of β-cells following short-term HFD feeding. Type I IFN signaling has been strongly associated with development of T1DM (32, 63), but less well described in T2DM. We also found a fingerprint of these inflammatory processes being active and enriched in human β-cells in prediabetes and in both T1DM and T2DM, which suggests that activation of inflammatory responses in human β-cells may represent an important step in the transition from functional compensation to decompensation in early stages of insulin resistance. It remains unknown if the inflammatory signal we have identified in mice is conserved across species, or if other signals drive the inflammatory response in humans.

In conclusion, we have identified a heterogenous interferon-associated immune response as a key early event in HFD, driving inflammatory processes in pancreatic β-cells that may negatively impact cellular plasticity. These findings highlight the need for fine-grained, time-resolved investigations to dissect the early transcriptional mechanisms induced by HFD, as it remains unclear whether these inflammatory processes persist, intensify, or resolve beyond 3 wk, and whether they ultimately exert detrimental or protective effects on cellular and metabolic health.

## Limitations

A limitation of this study is the low number of biological replicates used for transcriptomic analyses, particularly at the late time point for LFD (3 wk, n = 1). A low number of replicates limit statistical power and the ability to fully capture inter-individual heterogeneity, especially under HFD conditions, which mice are responding differently to in terms of weight and metabolic response. In this study, the mice used for joint snATAC-seq and snRNA-seq profiling were not outliers with respect to either absolute body weight or relative weight gain relative to a larger cohort of 69 LFD and 72 HFD mice, indicating that the transcriptomic data were not derived from extreme responders (Fig S1A and B). Furthermore, principal component analysis performed separately for each islet cell type showed robust separation by cell identity along PC1 (53–77% of explained variance), with additional segregation of samples by dietary condition along PC2 (13–19%), despite limited replication and inclusion of multiple time points within the LFD reference group. Finally, no statistical comparisons were made against the single 3-wk LDF sample in isolation. Instead, it was included as part of the pooled LFD reference group in all analyses, ensuring that no statistical analysis was supported by less than 2 individual mice. Together, these observations support the presence of reproducible diet-associated transcriptional signals while underscoring that the findings should be interpreted as exploratory and require validation in large cohorts.

## Materials and Methods

### Mice

8-wk-old C57BL/6J male mice from Taconic Bioscience (C57BL/6JBomTac; B6JBOM-M) were kept in IVC-cages, up to 4 mice per cage, and maintained in a 12-h light–dark cycle, at room temperature of 22 ± 3°C and ∼55% humidity with ad libitum access to food and water. The mice were acclimatized for 3 wk and fed LFD (D12450J; Research Diets) before experiment start. Hereafter, at day 0, the 11-wk-old mice were divided into three groups. One group continued the LFD as a control, and one group were fed either HFD (D12492; Research Diets) for 1 wk or 3 wk. At the end of feeding, the mice were used for several studies, including phenotypically characterization, Single-cell Multiome ATAC + Gene expression sequencing, and immunofluorescence microscopy as described below.

### Weight gain

Body weight was measured for all mice at baseline (week 0) and at 1 and 3 wk after diet initiation. Weight gain was calculated as the percentage change from mean baseline weight for each animal, using the formula: ((weight at timepoint − mean weight at week 0)/mean weight at week 0) × 100. This approach normalized weight gain to the cohort’s average starting weight, allowing for comparison across dietary groups and timepoints.

### Outlier analysis

Outliers in body weight and weight gain measurements were identified using the identify_outliers function (default parameters) from the rstatix package (version: 0.7.3) (64) in R (Version: 4.4.3). Observations were flagged as outliers if they deviated more than 1.5 times the interquartile range (IQR) below the first or above third quartiles. Outlier detection was performed separately for each measurement variable (body weight at weeks one and three, and weight gain at weeks one and three) to identify potential data quality issues or biological outliers. Identified outliers were retained in the analyses but annotated for visualization purposes to distinguish them from non-outliers.

### Intraperitoneal glucose tolerance test

After 1 wk or 3 wk, the glucose tolerance of the mice was assessed by intraperitoneal glucose tolerance test (IPGTT). Mice were fasted for 4 h in the morning and fasting plasma blood glucose levels were determined (T = 0). D-(+)-Glucose (G8270; Sigma-Aldrich) was administered intraperitoneally (2 g/kg) in a 0.9% saline suspension (420079; B. Braun) and plasma blood glucose levels were measured at 15, 30, 60, and 120 min after glucose injection using a glucometer (Cat#70919-70; FreeStyle Freedom Lite).

### Intraperitoneal insulin tolerance test

After 1 wk or 3 wk, the insulin tolerance of the mice was assessed by intraperitoneal insulin tolerance test (IPITT). Mice were fasted for 4 h in the morning and fasting plasma blood glucose levels were determined (T = 0). Insulin (A10AB01; Actrapid) in 0.9% saline suspension (420079; B.Braun) was administered intraperitoneally (0.75 U/kg) and plasma blood glucose levels were measured at 15, 30, 60 and 120 min after insulin bolus using a glucometer (70919-70; FreeStyle Freedom Lite).

### Fasting serum insulin

After 1 or 3 wk, the mice were fasted for 4 h in the morning. Tail or hindleg blood samples were collected and allowed to clot at room temperature for 30 min, whereafter the samples were centrifuged for 20 min at 2,000*g* at 4°C. The serum supernatant was extracted and used for insulin quantification using the Ultrasensitive Mouse Insulin ELISA kit (90080; Chrystal Chem) according to the manufacturer’s instructions.

### Estimating insulin sensitivity and β-cell function

We used a mathematical model of insulin-glucose homeostasis called SPINA (Structure Parameter Inference Approach)-Carb (19) to estimate two key parameters: insulin receptor gain (SPINA-GR), which measures insulin sensitivity, and β-cell insulin secretory capacity (SPINA-Gβ), which measures β-cell function. Both parameters were calculated using an implementation of the SPINA-Carb model (Version 5.0.0) (65) in an R statistical environment. The calculations relied on steady-state fasting concentrations of serum insulin (pmol/liter) and blood glucose (mmol/liter), employing the functions SPINA.GR for insulin receptor gain and SPINA.GBeta for β-cell secretory capacity.

### Statistical analysis

Data from phenotyping experiments: IPIGTT, IPITT, fasted blood plasma glucose, fasted serum insulin, body weight gain, SPINA-GR, and SPINA-Gβ were analyzed using the nonparametric Wilcoxon test to compare two means.

All code and parameters used for this analysis are available on GitHub (https://github.com/madsen-lab/islets_multiome_phenotyping). All analysis was carried out in Rstudio (version 4.4.0).

### Islet isolation

Mice used for islet isolation were euthanized using cervical dislocation. The pancreas was injected through the common bile duct with 3 ml dissociation buffer (0.7 mg/ml Collagenase P [Lot#33768602; Merck], 1X HBSS [14065049; Thermo Fisher Scientific] and 0.3 g/liter NaHCO3 [S5761; Sigma-Aldrich]), using a 30G syringe. The tissue was dissociated in a tube with 2 ml dissociation buffer for 15 min in a 37°C water bath. To further dissociate the tissue, the tubes were shaken gently by hand for 1 min. The dissociation was quenched by adding cold quenching buffer (1X HBSS, 0.3 g/liter NaHCO3 and 10% heat-inactivated fetal bovine serum [VIA] [B15-005; Biochrom]) to a total volume of 20 ml.

The tissue solution was centrifuged for 2 min at 150*g* at room temperature (RT) in a swing-bucket centrifuge (Acceleration = 8, brake = 2), the supernatant was aspirated until there was 10 ml left. This wash step was repeated two times. The tissue was further dispersed by pipetting the tissue solution through a 14G venflon (without the needle) 5 times. Hereafter, the tissue solution was spun down for 7 min at 200*g* at RT, supernatant removed, and tissue pellet was resuspended in 10 ml of a histopaque density gradient used to separate islets from non-endocrine tissue (5:11 Histopaque-1077 [10771; Sigma-Aldrich] and 6:11 Histopaque-1119 [11191; Sigma-Aldrich]). The Histopaque solution was centrifuged for 20 min at 380*g* at RT (Acceleration = 8, break = 2) in 15 ml tubes. The islet containing supernatant was aspirated and the supernatant was filtered through a 400 μm filter (43-50400-03; pluriS-trainer), followed by reversed filtering using a 70 μm filter (11597522; Fisherbrand). Islets were hand-picked two times and contained in RPMI 1640+ GlutamaxTM medium (61870044; Gibco) (11 mM glucose) supplemented with 5% heat-inactivated fetal bovine serum (B15-005; Biochrom) and 100 U/ml penicillin and 100 mg/ml streptomycin (DE17-602E; Lonza).

### Nuclei isolation from islets

We adapted the 10x Genomics protocol (CG000375; Demonstrated Protocol_NucleiIsolationComplexSample_ATAC_GEX_Sequencing_RevB) (66) for isolation of nuclei from isolated islets. Detergent-based permeabilization of the cell membrane was used for nuclei isolation. Freshly isolated islets were moved to biomasher II Eppendorf tubes and centrifuged for 5 min at 400*g* in a swing-bucket centrifuge at 4°C. The media-supernatant was carefully removed, leaving ∼ 20 μl media on top of the islet pellet. To release the nuclei from the islets, 100 μl nuclei preparation buffer (NBP) (10 mM HEPES pH 7.5 [H3375; Sigma-Aldrich], 1.5 mM MgCl_2_ [M8266; Sigma-Aldrich], 40 mM KCl [P9541; Sigma-Aldrich], 250 mM sucrose [S0389; Sigma-Aldrich], 0.5 mM Spermidine [S2626; Sigma-Aldrich], 1% bovine serum albumin [BSA] [M0314L; Merck], 0.1% IGEPAL CA-360 [I8896; Sigma-Aldrich], 0.2 mM DTT [B1034A; NEB] and 1 U/μl RNase inhibitor [M0314; NEB] dissolved in DEPC water) was added to the islet pellet and the pellet was homogenized 20 × times using the Biomasher II Disposable Micro Tissue Homogenizer (EOG-sterilized) (320103; NIP). Hereafter, 1,000 μl NBP was added to the tube. The solution was incubated on ice for 5 min, gently pipetting halfway using wide-bore pipette tips. To remove cell debris, the nuclei suspension was passed through a 70 μm tip strainer into a 1.5 ml DNA low bind Eppendorf tube. To increase the yield of nuclei the biomasher Eppendorf tube was washed with 500 μl NPB, and passed through the tip strainer, into the same 1.5 ml tube. The nuclei suspension was centrifuged at 500*g* in a swing-bucket centrifuge for 5 min at 4°C and the NPB supernatant was removed until there was ∼50 μl left. The nuclei suspension was washed by adding 1,000 μl 2% BSA in 1X phosphate-buffered saline (PBS) + 1 U/μl RNase inhibitor to the nuclei suspension (not mixed!) and after 5 min on ice, the suspension was mixed to resuspend the nuclei pellet. Hereafter, the nuclei suspension was centrifuged at 500*g* in a swing-bucket centrifuge for 5 min at 4°C, and the BSA supernatant was removed.

To permeabilize the nuclei membrane for transposition the nuclei were incubated in 100 μl 0.1X lysis buffer (10 mM Tris–HCl pH 7.5 [103156X; VWR], 3 mM MgCl2, 10 mM NaCl [S3014; Sigma-Aldrich], 0.01% Tween-20 (P1379; Sigma-Aldrich), 0.01% IGEPAL CA-630, 1% BSA, 0.001% Digitonin (BN2006; Thermo Fisher Scientific), 1 mM DTT, 1 U/μl RNase inhibitor dissolved in DEPC water). After 2 min, the permeabilization was stopped by diluting the lysis buffer with 1 ml Wash buffer (10 mM Tris–HCl pH 7.5, 3 mM MgCl2, 10 mM NaCl, 0.1% Tween-20, 0, 1% BSA, 1 mM DTT, 1 U/μl RNase inhibitor dissolved in DEPC water). The solution was mixed by careful pipetting 5 times. Samples were centrifuged at 500*g* in a swing-bucket centrifuged for 5 min at 4°C and the wash buffer supernatant was removed. The nuclei pellet was resuspended in 60 μl 1X nuclei buffer (10x Genomics), (1 mM DTT and 1 U/μl RNase inhibitor dissolved in DEPC water). Finally, any nuclei aggregates were removed by passing the nuclei solution through a 40 μm tip strainer into a new DNA low bind Eppendorf tube, and the nuclei suspension was centrifuged at 500*g* in a swing-bucket centrifuge for 5 min at 4°C and the supernatant was removed until there was 7 μl nuclei buffer left.

For counting, 1 μl nuclei solution was diluted in 10 μl 1X nuclei buffer and 9 μl trypan blue dye (Cat#1450021; Bio-rad). Nuclei were counted using a Bürker pattern counting chamber (BR718920; Sigma-Aldrich). 5,000-16,000 nuclei were submitted for Single-cell Multiome ATAC + Gene expression sequencing.

### Single cell multiome ATAC + gene expression sequencing

We used the Single-cell Multiome ATAC + Gene expression sequencing technique provided by 10X Genomics, to simultaneously detect changes in gene expression (snRNA-seq) and chromatin accessibility (snATAC-seq). This approach was applied to nuclei from islets of Langerhans isolated from seven mice (143–300 islets/mouse) fed with either LFD (n = 3) or HFD for either 1 wk (n = 2) or 3 wk (n = 2). Each mouse sample was processed separately.

Isolated nuclei were prepared for transposition and library generation using the following 10x Genomics kits: Chromium Next GEM Single Cell Multiome GEM Kit A, 16 rxns (PN-1000232), Chromium Next GEM Single Cell Multiome Amp Kit A, 16 rxns (PN-1000233), Chromium Next GEM Single Cell Multiome ATAC Kit A, 16 rxns (PN-1000280), and Library Construction Kit, 16 rxns (PN-1000190).

The samples were prepared in accordance with the manufacturer’s instructions (CG000338; Chromium Next GEM Single Cell Multiome ATAC + Gene Expression User guide). Libraries were sequenced on the Illumina NovaSeq 6000 System (20012850; Illumina).

### Single cell multiome ATAC + gene expression sequencing analysis

#### Demultiplexing

Raw base call files were demultiplexed into FASTQ files using the mkfastq pipeline in 10X Genomics Cell Ranger ARC (version: 2.0.1).

#### Generation of count matrices

All code and parameters used for this analysis are available on GitHub (https://github.com/madsen-lab/islets_multiome_analysis). All analysis in this section, except for alignment and SCENIC+, was carried out in R (version 4.3.0).

snRNA-seq data were aligned to the mm10 (GRCm38) reference genome with STARsolo (version: 2.7.9a) (67), counting both intronic and exonic reads or only exonic reads (soloUMIlen = 12, soloCellFilter = none, soloType = CB_UMI_Simple, soloCBmatchWLtype = 1MM, clipAdapterType = CellRanger4, soloFeatures = GeneFull_Ex50pAS Gene). The snATAC-seq data were aligned to the mm10 reference genome using cellranger-arc count (default parameters) from 10X Genomics Cell Ranger ARC (version: 2.0.1) (68).

#### Quality control

The snRNA-seq count matrix was filtered for high-quality barcodes using valiDrops (version: 0.1.0) (69) functions rank_barcodes, quality_metrics, and quality_filter (default parameters). In addition, we added a global filter for the mitochondrial fraction < 0.025 and exon ratio > 1. The snATAC-seq count matrix was filtered using ArchR (version: 1.0.2) (70) (TSSEnrichment ≥15, BlacklistRatio ≤ 0.05, nFrags ≥ 2,500). Doublets were identified in both modalities using DoubletFinder (version: 2.0.3) (71) for the RNA modality and ArchR for the ATAC modality. Nuclei which passed quality control in both modalities were retained. Nuclei called as doublets in both modalities were removed.

#### Dimensional reduction and cluster identification

WNN analysis (version: 4.4.0) (20) was used to generate a single representation of the combined snRNA and snATAC-seq data. WNN analysis requires two dimensional reductions, one for each modality.

Dimensional reduction for the snRNA-seq data was computed in seurat workspace. Count normalization, variable feature selection, dimensional reduction and batch integration were performed simultaneously using JOINTLY (version: 1.0) (default parameters) (72). snRNA-seq counts were normalized with Seurat’s LogNormalize function (scale.factor = 1e4).

Before processing the snATAC-seq data, the snRNA-seq data were used to further identify any remaining doublets based on poly-hormone expression of the major endocrine marker genes: *Ins2*, *Gcg*, *Sst*, and *Ppy*. Using a deep clustering approach (resolution = 20), clusters were identified as potential doublets (polyhormonal clusters) if they had an average scaled expression above 0.6 for more than one of marker gene with a sum of scaled expression values exceeding 1. These were removed from both modalities.

Hereafter, a dual-fold methodology was employed to compute batch-corrected dimensional reductions for the snATAC-seq data. Variable feature selection and integration was performed using 5,000 bp window tiles. The top 150,000 most variable windows were identified using ArchR’s Iterative latent semantic indexing, with 4 iterations, these were used for integration with LIGER (version: 1.0.0) (73) (default parameters). The resulting embeddings were used to cluster the snATAC-seq data (20 dimensions, resolution = 2), for putative cluster annotation which was used for peak detection. The clusters were annotated by evaluating the gene score (a prediction of gene expression based on the accessibility of regulatory elements in the vicinity of the gene) and gene expression of major marker genes. We used the imputed weight method MAGIC on the gene scores for reducing noise of the snATAC-seq data. The identified clusters were subsequently used for reproducible peak calling using MACS2 in ArchR (74, 75 *Preprint*, 76) (minCells = 20, maxCells = 5,205, minReplicates = 2, maxReplicates = 3). Lastly, the generation of batch-corrected dimensional reductions using LIGER was repeated as described above but now using the top 150,000 variable peaks instead of windows.

Meta data information, peak matrix, gene scores and LIGER embeddings from the ArchR object were transferred to the Seurat object containing the snRNA-seq data using ArchRtoSignac (version: 1.0.3) (77). From here, analysis was performed using the combined data in the Seurat object unless otherwise specified.

A WNN-graph was constructed using both modalities (rna embeddings = 1:15, peak embeddings = 1:20) and used for graph-based clustering as well as Uniform Manifold Approximation and Projection (UMAP) visualization. Cell type identities were manually assigned to cell clusters by evaluating expression of prior knowledge cell-type markers.

#### Evaluation of batch mixing

Batch mixing of replicates before and after integration was evaluated for each diet condition in both modalities. This evaluation was conducted using unintegrated snRNA-seq principal component (PC) embeddings (1:15 dimensions) and snATAC-seq singular value decomposition (SVD) embeddings (1:20 dimensions), as well as integrated snRNA-seq JOINTLY embeddings (1:15 dimensions) and snATAC-seq LIGER embeddings (1:20 dimensions).

The assessment employed the local inverse Simpson’s index (LISI) (78) and the average silhouette width (ASW). Integration LISI (iLISI) and batch ASW (bASW) were calculated using these embeddings with the evaluateEmbedding function from http://www.github.com/madsen-lab/JOINTLY_reproducibility (72).

Both metrics were rescaled as previously described by Luecken et al (79). For iLISI, a value of 0 indicates low batch integration, while a value of 1 indicates high batch integration. For bASW, a value of 0 reflects low cell type separation and high batch separation, whereas a value of 1 indicates optimal performance with high cell type separation and low batch separation.

### Cell type prioritization

To identify the cell type in which transcription was the most perturbed by HFD feeding, we performed cell type prioritization analysis using Augur (version: 1.0.3) (23) using the calculate_auc function (subsample_size = 12, n_subsamples = 150). The most perturbed cell type is the cell type with the highest area under the curve (AUC) exceeding 0.5.

### Differential gene expression analysis

Differential gene expression analysis was performed using the DESeq2 package (version: 1.40.2) (80) between condition and clusters (including subclusters), using aggregated (pseudo bulk) counts from each cell type per replicate obtained with Edgers Seurat2PB function (version: 3.42.4) (81) (default parameters). The DESeq function (default parameters) was used to estimate size factors, count normalization and calculating differential gene expression based on a negative binomial distribution. Comparisons across diet were performed using the Likelihood Ratio Test (LRT) to identify genes, which change across time points, i.e., genes with a dynamic expression pattern. Pairwise comparisons between overall cell type clusters as well as between subclusters were performed using a paired Wald test, specifying which condition each replicate is from. Genes were filtered based on their adjusted *P* value (FDR ≤ 0.05).

### Fuzzy clustering of genes

To explore the temporal regulation of gene expression, all genes with a dynamic expression pattern (i.e., those changed in any time point across conditions, identified in the DESeq2 LRT analysis), were clustered based on normalized pseudo bulk gene expression averaged by condition, using fuzzy clustering with the package MFuzz (version: 2.60.0) (82). First, expression values were standardized using the standadise function (default parameters), and the optimal fuzzifier was estimated using mestimate (default parameters). Hereafter, the optimal number of clusters for the identified fuzzifier was performed by calculating the minimum distance between 1 to 20 clusters by using the function Dmin (crange = seq (2,20,1)), repeats = 15). The optimal number of clusters was defined as the number of clusters at which the minimum centroid distance begins to decrease slowly. Finally, clustering was performed with mfuzz (default parameters). The genes in each cluster were used for pathway analysis.

### Pathway analysis

Pathway analysis was performed to explore the functional relevance of differential expressed genes. The analysis was performed using the function enrichGO (ont = “BP,” pvalueCutoff = 0.2, qvalueCutoff = 0.2) from the ClusterProfiler package (version: 4.8.3) (83). Mouse genes were annotated using the org.Mm.e,g.db package (version: 3.17.0) (84). GO-terms for biological processes were used as reference gene sets, and all genes which were used as input for the DESeq2 analysis were used as background (universe). Gene sets with an FDR ≤ 0.05 were considered significantly enriched.

### eRegulon identification

In Python (version: 3.8.17), eRegulon interference analysis in β-cells across conditions was performed with SCENIC+ (version: 1.0.1.dev4+ge4bdd9f) (30). Raw snRNA-seq counts, peak matrix, and condition annotations (for nuclei within the β-cell cluster) were extracted from the seurat object and used for the analysis. First, PyCisTopic (version: 1.0.3.dev18+ge563fb6) (85), within the SCENIC+ framework, was used to perform latent Dirichlet allocation topic modeling on the peak matrix (n_itr = 500, alpha = 50) to identify sets of co-accessible regions (topics). Subsequently, PyCisTarget (version: 1.0.3.dev1+g3fde1ce) (30), within the SCENIC+ framework, was used to identify differentially accessible regions (DARs) using a Wilcoxon rank-sum test (default parameters) and to identify enriched motifs within the topics and DARs (default parameters). Motif enrichment analysis was carried out using the clustered motif collection database from SCENIC+ (v10, mm10). The results, together with the raw snRNA-counts, were used as input to the SCENIC+ algorithm (default parameters) against a list of all known mouse transcription factors provided by SCENIC+. Identified putative eRegulons were then employed for downstream analysis.

### Identification of high-quality eRegulons

High-quality eRegulons were identified through correlation analysis of semi-pseudo-bulked data per condition (100 meta-cells per condition, each with 10 cells) using Pearson correlation. The correlation analysis was performed using the min–max scaled product of transcription factor gene expression and of transcription factor motif activity with activity scores for target genes and enhancers from SCENIC+ (computed using AUCell (86)), for each eRegulon. This was to ensure that transcription factors were active in cells where their target genes were expressed, and target regions were accessible (correlation >= 0.6).

Motif activity analysis, used in the correlation analysis, was performed using chromVar (version: 1.22.1) (87) (default parameters) within the ArchR framework, using the motif collection database from SCENIC+. Subsequently, the motif activity for each transcription factor was calculated by averaging the deviation score, from chromVar, for each motif associated with the specific transcription factor.

### Curation of phenotype-associated gene sets

Phenotype-associated gene sets were derived from the Attie lab diabetes database (http://diabetes.wisc.edu/correl_f2.php, access: November 15th, 2023) (36), who has performed correlation analysis between diabetes-related clinical phenotypes and gene expression within islets of Langerhans, from obese B6:BTBR F2 mice. For each clinical phenotype, all genes with a Pearson correlation coefficient >0.1 (positive correlation) or <−0.1 (negative correlation), were used to create gene sets. Phenotypes categorized under “Metabolism and Energy Regulation” and “Inflammation and Immune Response.” as kept.

### Cell-cell communication analysis

To infer cell-cell communication between β-cells and all other cell types across conditions (LFD, 1- and 3-wk HFD), we used the MultiNicheNet package (version: 1.0.3) (40 *Preprint*), to predict ligand-receptor interactions between all cell types (senders) and β-cells (receivers) between conditions. First, MultiNicheNet uses aggregated pseudo bulk counts and the DESeq2 package for differential gene expression between conditions. Differential expressed ligands, receptors, and target genes were identified using the thresholds: logFC > 1 and adjusted *P*-value ≤0.05. The ligand activity within receivers was calculated using the top 250 target genes with the highest regulatory score (this score reflects how much prior knowledge supports specific ligand – target gene regulation). Ligand-receptor interactions were prioritized, within each condition, using a weighted combination of min–max scaled scores of the following criteria as recommended by the developers: (1) upregulation of the ligand in the senders and upregulation of the receptor in the receiver at the condition of interest (weight = 1), (2) sufficient expression levels of the ligand and receptor in samples of the same group (weight = 1, to mitigate influence of outliers), (3) cell type and condition specific expression of the ligand in the sender, and receptor in the receiver (weight = 2, to mitigate the influence of upregulated ligand/receptors that are weakly expressed), and lastly, (4) high ligand activity (weight = 2). Based on this combined score, the top 30 ligand-receptor interactions, for each condition, were visualized in a circos plot. Target genes of ligand-receptor pairs were identified by calculating Pearson and Spearman correlation coefficients between the min–max scaled product of pseudo bulk expression of the ligand and receptor of interest and the expression of the target gene. Target genes with a correlation coefficient greater than 0.5 or less than −0.5 were used for downstream analysis.

### Overrepresentation analysis and gene set enrichment analysis

Overrepresentation analysis of gene sets was performed by constructing a 2 × 2 contingency table. This analysis aimed to explore the potential association between gene sets, denoted *S* (such as target genes identified in cell-cell communication analysis) and eRegulon target genes, denoted D, and *N* is the total number of genes (Table 1). Here *N*_*DS*_ is the total number of genes in both *D* and *S*, *N*_*D*_*-N*_*DS*_ is the number in genes that are in *D* but not in *S*, *N*_*S*_*-N*_*DS*_ is the number of genes that are in *S* but not in *D*, and *N-(N*_*D*_*+N*_*S*_*-N*_*DS*_*)* is the number of genes that are in neither *D* nor in *S*. Fisher’s exact test was employed to determine if there was a significant association between *D* and *S*. Significant associations was determined based on adjust *P*-values (FDR ≤ 0.05).

**Table 1.** Contingency table used for overrepresentation analysis of gene sets.

​	In *S*	Not in *S*
In *D*	*N*_*DS*_	*N*_*D*_ − *N*_*DS*_
Not in *D*	*N*_*S*_ − *N*_*DS*_	*N* − (*N*_*D*_ + *N*_*S*_ − *N*_*DS*_)

Genes in the analysis are cross classified by membership in the query gene set *S*, and in the eRegulon target gene set *D*. *N* is the total number of genes. *N*_*DS*_ is the total number of genes in both *D* and *S*, *N*_*D*_ *− N*_*DS*_ is the number in genes that are in *D* but not in *S*, *N*_*S*_ *− N*_*DS*_ is the number of genes that are in *S* but not in *D*, and *N − (N*_*D*_ *+ N*_*S*_ *− N*_*DS*_*)* is the number of genes that are in neither *D* nor in *S*.

GSEA was performed using clusterProfiler (version: 4.8.3) (83) (default parameters). Gene lists used for ranking and testing are indicated in Figure legends.

### Gene module activity

A ranked based methods UCell (version: 2.4.0) (88) (maxRank = 1,000) was used to quantify the activity of gene sets that were not calculated in SCENIC+ (i.e., eRegulon target genes and enhancers). UCell calculates activity scores by ranking all genes (up to the maximum rank) expressed in each individual cell based on their expression levels, where a lower rank indicates greater expression. For a gene set of interest, UCell performs a Mann–Whitney *U* test, with the activity score represented as the normalized *U*-statistic, which ranges from 0 to 1. A score of 1 indicates that the genes in the gene set are among the most highly expressed in the cell, while a score of 0 indicates that these genes are among the least expressed.

### Inflammatory response and UPR subcluster identification

For the identification of β-cells with a high inflammation, the TNF and IFN-β target genes identified by MultiNicheNet, were used to define an “Inflammatory response” gene set. For the high UPR subcluster, a gene set was assembled from the Gene Ontology biological process terms for ATF6-, IRE1-, and PERK-mediated unfolded protein response, retrieved for Mus musculus from MSigDB collection C5 via the msigdbr R package (version 7.5.1), and combined into a single non-redundant set. Subsequently, UCell activity scores were computed for each β-cell, following the procedure outlined in the section “Gene Module Activity.”

For UPR subcluster identification, cells with a score at or above the 75th percentile of the score distribution in LFD β-cells were classified as high UPR (≥0.102). For inflammatory response subcluster identification, we joint threshold in HFD by sorting by finding the inflection point using the inflection package (version: 1.3.6) (89) (default parameters) on the empirical cumulative distribution of the activity scores for β-cells from the HFD week one or HFD week three in a descending order. β-cells with an activity score exceeding the inflection point (>0.106) were annotated as belonging to a high inflammatory subcluster.

### In vivo validation of high inflammatory response β-cells

To validate the presence of high inflammatory response β-cells identified bioinformatically in mice fed a HFD for 3 wk, we performed immunofluorescence staining of pancreatic tissue from mice fed either an LFD or HFD for 3 wk (n = 4 per diet). Two slides per mouse were stained, each containing two tissue sections. Insulin was used as a β-cell marker and to define islet regions, STAT1 as a marker of high inflammatory response β-cells, and DAPI for nuclear staining.

#### Tissue preparation

Dissected pancreatic tissues were fixed in 4% paraformaldehyde (PFA) in 1× PBS (pH 7.4) at 4°C for 5 h. Tissues were rinsed three times for 15 min in cold 1X PBS at 4°C, followed by cryoprotection in 30% sucrose in 1XPBS overnight at 4°C. Tissues were then cryoembedded in O.C.T. (NC1862249; Sakura Tissue-Tek) on dry ice, with 70% ethanol sprayed around the mold to accelerate freezing and stored at −80°C until sectioning.

For sectioning, a cryostat (CM1860; Leica) was precooled to −21°C for 6 h before use. Cryoblocks were equilibrated to the chamber temperature for 30 min before mounting onto the cutting disk and for an additional 30 min after mounting. Tissues were sectioned at a thickness of 10 μm (100 μm apart) and mounted on slides, with two sections per slide. Slides were air-dried at RT for at least 1 h, for good tissue adherence.

#### Immunofluorescence staining

Frozen slides were removed from −80°C storage, thawed for 5–10 min, and submerged in 1X PBS. Slides were washed for 10 min in 1X PBS on a shaking table to dissolve the O.C.T., followed by antigen retrieval for 1 h at 37°C in citrate buffer (9.5 mM citric acid and 41.5 mM sodium citrate in Milli-Q water). Slides were washed 3 × 5 min in 1X PBS and assembled with a cover plate (72-110-017; Epredia) on a Sequenza rack (73-310-017; Epredia). Sections were permeabilized in 0.15% Triton X-100 in 1X PBS for 1 h at RT, followed by 3 × 5-min washes in 1X PBS. Sections were then blocked in 1% BSA in PBST (1X PBS with 0.1% Tween-20) for 2 h at RT.

Slides were incubated overnight at 4°C with primary antibodies against STAT1 (1:50; ab239360; Abcam; rabbit) and insulin (ready-to-use; IR00251-2; Agilent; guinea pig). Following 3 × 5-min washes in 1X PBS, slides were incubated with secondary antibodies for 1.5 h at RT protected from light: Alexa Fluor 488-conjugated anti-guinea pig (1:200; ab150185; Abcam) and Alexa Fluor 546-conjugated anti-STAT1 (1:250; A-11035; Invitrogen). Slides were washed 3 × 5 min in 1X PBS. Nuclei were stained with DAPI (4′,6-diamidino-2-phenylindole; 1 μg/ml; 10116287; Thermo Fisher Scientific) diluted in 1% BSA in PBST for 5 min at RT protected from light, followed by 3 × 5 min washes in 1X PBS.

Finally, slides were mounted using Vectashield antifade mounting medium (H-1400-10; Vector Laboratories), sealed with nail varnish, and allowed to dry for several hours protected from light. Mounted slides were stored at 4°C for several days or at −20°C for long-term storage.

### High throughput image acquisition

Images were acquired on an automated Nikon Ti2 widefield microscope equipped with a Teledyne Photometrics Kinetix sCMOS camera (20.8 × 20.8 mm FOV) and an Andor Zyla 5.5 sCMOS camera, controlled using a custom semi-automated imaging workflow built in the JOBs module of the NIS-Elements software (Version: 6.10.01).

DAPI (DNA), FITC (insulin), and TRITC (STAT1) fluorophores were excited sequentially using the 400, 460, and 525–660 nm LEDs, respectively, from a pE-300 light source. Nikon BL series fluorescence filter cubes DAPI, GFP/FITC, and TRITC/Cy3 were used for the fluorophore set.

#### Low-magnification overview and manual tissue outline

Each slide was initially rapidly scanned in brightfield using a 4X objective in a 20 × 9 tile grid (7.52 × 3.42 cm) with 15% tile overlap. This overview image was used to locate the pancreatic tissue on the slide and allowed the user to manually create approximate outlines of the tissue regions.

#### Focus surface generation

To account for variation in sample height across the slide, a small set of focus points (positioned at the corners and center) was automatically defined for each tissue region. At each point, a DAPI fluorescence-based autofocus routine was performed across a 300 μm search range in two steps (30 μm coarse steps, followed by 3 μm fine steps) to determine focus heights of the tissue. The resulting Z-positions were used to construct an interpolated and continuous focus surface, allowing a subsequent fast and efficient complete scanning of all tissue regions.

#### Islet detection

Tile-scans (4× objective, 15% tile overlap) of FITC fluorescence were now acquired for each tissue region and subjected to on-the-fly image analysis to automatically identify insulin-positive regions (hereafter referred to as islets) within each tissue region. The analysis combined low pass filtration (4×) and intensity thresholding to generate initial binaries, which were further refined by hole filling, smoothing (6×) and morphological closing using a convex hull operation. Very small, detected islets (<30 μm) and islets touching scan borders were filtered out and the islets were subsequentially dilated by 100 μm to merge islets in close proximity. Together this ensured that a comprehensive list of unique individual islet regions was efficiently created for the following automated imaging at higher resolution.

#### High-resolution multichannel imaging

The JOBs workflow then switched to a 20× objective and an optical configuration for higher resolution imaging of each automatically detected islet. For the first islet in each tissue region, a long-range autofocus was used (400 μm range, 30 μm step). All subsequent islets used a finer, faster autofocus routine (90 μm range; 5.4 μm coarse step, 1.8 μm fine step) performed in the center of each islet before acquisition. Islets located near one another were merged and captured within a single image, as previously described. Each islet was tile-scanned (15% overlap, stitched using blending) with a 20X objective using a three-channel (DAPI, FITC, and TRITC) widefield epifluorescence configuration to create a single composite image per islet/islets.

In total, 16 slides from 8 mice (50–100 high-quality images and 64–149 islets per mouse) were acquired for the downstream analysis.

### Image analysis

Immunofluorescence microscopy images were provided in a Nikon ND2 format containing three fluorescent channels as described above. All acquired images were manually inspected to assess image quality. Images that lacked clearly visible islet structures or were out of focus, were excluded from the downstream analysis. Image preprocessing and feature extraction was performed in python (version: 3.13.12) using the Scikit-image (version: 0.26.0) (90) and SciPy’s ndimage (version: 1.18.0) (91) libraries. Downstream analysis was performed in R (version: 4.5.3).

#### Background correction

Images were loaded using the nd2 (version: 0.11.3) (92) library. To remove non-specific background fluorescence, we estimated and subtracted a background signal from each channel using an iterative smoothing approach. For each channel, we started with a copy of the raw image and repeatedly smoothed it using a local average filter (15-pixel window). After each smoothing step, we kept the lower of the two values at every pixel, the smoothed value or the original raw value, so that the background estimate could never exceed the observed signal. This process was repeated for up to 300 iterations, or until the estimate stopped changing substantially (defined as a mean absolute change of less than 0.05 between iterations). The resulting background estimate was then subtracted from the raw image to produce a background-corrected image for each channel.

#### Islet segmentation

To identify islet regions within background-corrected insulin images, we used Otsu thresholding as an initial coarse segmentation. The resulting binary masks were refined using morphological closing (disk structuring element, radius = 10 pixels) to smooth boundaries and connect fragmented regions, followed by hole filling. Small objects with a maximum area of 10,000 pixels were removed to exclude very small insulin-positive areas. Individual insulin-positive areas, now called islets, were then identified using connected component labeling of the final binary mask.

#### Nuclei segmentation and filtering

Cell nuclei were segmented from the background-corrected DAPI channel. Otsu thresholding was first applied to separate nuclear signal from background, producing a binary mask of nucleus-positive pixels. Because adjacent nuclei can form connected regions in the binary mask, a watershed-based splitting step was used to separate them. A Euclidean distance transform was applied to the mask, assigning each pixel a value corresponding to its distance from the nearest background pixel. Local maxima in the resulting distance map, separated by a minimum of 7 pixels, were used as seed points, to mark individual nuclear centers. Watershed segmentation was then applied to the distance map, expanding each seed outward until reaching the mask boundary or an adjacent expanding region, thereby splitting connected nuclei. Small objects with areas of <30 pixels were removed. Each remaining nucleus was expanded outward by up to 8 pixels to approximate the surrounding perinuclear cytoplasmic region, with expansion boundaries between neighboring nuclei set at the midpoint to prevent overlapping. Together these two regions make up segmented cells. Segmented cells were retained only if ≥80% of their area overlapped with islet areas. The remaining cells inside the islets were used for feature extraction.

#### Region definition

Three measurement regions were defined using the generated masks. (1) the islet regions (insulin mask), (2) nuclear regions (DAPI-positive inside the insulin mask), and (3) perinuclear cytoplasmic regions (expanded nuclear labels minus the nuclear mask inside the insulin mask).

#### Feature extraction

Feature extraction was performed at both region levels: the islet level and cell level. For each level, we measured background-corrected fluorescence pixel intensity statistics (mean, median, and standard deviation) for each channel (insulin, STAT1, and DAPI). To determine the spatial position of each cell within the islet, we created a distance map from the insulin mask using Euclidean distance transform. Using the insulin mask, we obtain how far every pixel inside the islet was from the nearest edge of the islet, generating a map where each pixel has a distance value. Pixels at the islet edge have a distance of zero, while pixels deeper inside the islet have progressively larger distance values. For each segmented cell, we calculated its centroid, which is the geometric center point of the nucleus determined by finding the average x and y coordinates of all the pixels that make up the cell. For each cell, we extracted the distance from its centroid to the islet edge, by looking up the x- and y-coordinates in the islet distance map to find the corresponding distance value. Islets with less than 10 cells were excluded from the analysis.

#### Identification of STAT1 high β-cells

To identify cells with elevated STAT1, percentile thresholds (70th, 75th, 80th, 85th, 90th, and 95th) were calculated separately for STAT1 mean pixel intensity (MPI) in nuclear and perinuclear cytoplasmic regions using the pooled distribution of all cells from both LFD and HFD mice. A cell was annotated as STAT1 high if its nuclear STAT1 MPI exceeded the corresponding nuclear percentile threshold, its perinuclear STAT1 MPI exceeded the corresponding perinuclear threshold, or both. To assess differences in STAT1 high cell abundance between diet groups, for each mouse we calculated the percentage of STAT1 high cells out of the total number of cells.

#### Spatial distribution of high and low STAT1 cells

Spatial enrichment of STAT1-high cells was assessed at the islet level by modeling the number of STAT1-high cells relative to the total number of cells within each spatial compartment (periphery or core). For each islet, cells were first assigned to spatial compartments based on centroid distance from the islet edge, with the closest 30% classified as periphery and the remainder classified as core.

For each islet and spatial compartment, we fit a binomial mixed-effects model using the glmmTMB package (version: 1.1.14) (93, 94), modeling the number of STAT1 high cells in a location, out of the total number of cells in that location. Location was included as a fixed effect, and islet identity included as a random effect to account for between-islet variation. Each islet corresponds to a unique mouse, slide, and imaging field. A mouse-level random effect was also evaluated but did not improve model fit and contributed negligible variance. Regression coefficients for location represent log-odds ratios (periphery vs. core). The log-odds ratios were exponentiated to obtain odds ratios, comparing the odds of a cell being STAT1 high in the periphery relative to the core. Statistical significance of the spatial effects was assessed using a permutation-based approach (999 permutations), in which STAT1 status labels were randomly shuffled within islets while preserving spatial locations labels. For each permuted dataset, the full modeling procedure was repeated and fixed-effect coefficients were extracted. Empirical two-sided *P*-values were calculated as the proportion of permutation-derived log-odds ratios with absolute values which are greater than or equal to the observed log-odds ratio, using a +1 correction in both numerator and denominator.

### Public single-cell RNA-seq data

To investigate whether inflammatory cytokines and nutrient stressors induce gene expression programs similar to those identified in short-term HFD-treated murine islets, we obtained publicly available scRNA-seq data of islets from non-diabetic human donors treated ex vivo with glucose (22 mM) and/or palmitate (0.5 mM), IL-1β (1 ng/ml) and IFNγ (1,000 U/ml), IFNα (2,000 U/ml), or untreated as control for 24 and 72 h (24, 25). Filtered barcode-, gene - count matrices, and a meta data file were available for download from GEO (GSE218316) and used to create a Seurat object. The Seurat object was subset to only include β-cells based on the provided annotation “Celltype.” These β-cells were used to calculate gene module activity scores using the five clusters of DEGs identified our dataset using fuzzy clustering as described in the section “Fuzzy clustering of genes” or for differentially gene expression between treated and untreated β-cells following 24 or 72 h of treatment using pseudo-bulked counts as described in the section “Differential gene expression analysis.” We filtered genes with an adjusted *P*-value (FDR ≤ 0.05) and a log2 fold change (log2FC ≥ 0.5) to retain those with significantly increased expression after treatment. To enable cross-species comparison, the murine gene symbols from each of the five gene clusters were converted to human orthologs by mapping mouse gene symbols to Entrez IDs using AnnotationDbi (version: 1.62.2) (95) and org.Mm.eg.db (version: 3.17.0) (96). We then matched these mouse Entrez IDs to human orthologs using Orthology.eg.db (version: 3.17.0) (97) and converted the human Entrez IDs to human gene symbols using org.Hs.eg.db (version: 3.17.) (84). Gene module activity scores were then calculated for each human β-cell using these human versions of the murine HFD-induced gene clusters, following the procedure outlined in the section “Gene Module Activity.”

Furthermore, to assess whether inflammatory gene patterns were relevant in human diabetes, we sourced scRNA-seq data from two studies: (1) Bandesh, K. et al 2025 (41 *Preprint*) and (2) the human pancreas analysis project (HPAP) (42), curated by Elgamal et al (98). (1) The Bandesh et al dataset comprises scRNA-seq data from human Islets of Langerhans, from non-diabetic individuals or individuals with prediabetes or T2DM. A processed seurat object called “Reintegrated transcriptomes of Beta pancreatic islet cells” was available for download at CELLXGENE (https://cellxgene.cziscience.com/collections/58e85c2f-d52e-4c19-8393-b854b84d516e, access: October 4th, 2024). (2) The curated HPAP dataset comprises an integrated map of scRNA-seq data from human Islets of Langerhans, from non-diabetic individuals or individuals with T2D or T1D. A processed and annotated seurat object containing this data was downloaded (https://www.gaultonlab.org/pages/Islet_expression_HPAP, access: June 6th, 2024), and subset to exclusively include β-cells based on the provided annotation “Cell Type Grouped.” These two datasets were used to calculate gene module activity scores of TNF and IFNβ putative target genes in each β-cell, following the procedure outlined in the section “Gene Module Activity.” The TNF and IFNβ target genes were converted from mouse to human gene symbols by mapping mouse gene symbols to Entrez IDs using AnnotationDbi (version: 1.62.2) (95) and org.Mm.eg.db (version: 3.17.0) (96). We then matched these mouse Entrez IDs to human orthologs using Orthology.eg.db (version: 3.17.0) (97) and converted the human Entrez IDs to human gene symbols using org.Hs.eg.db (version: 3.17.) (84).

### INTERFEROME database search

The INTERFEROME database (43) (v 2.0.1) (analysis run March 6th, 2025) is a manually curated database for interferon regulated genes, identified in transcriptomics studies. We submitted the upregulated genes from the high inflammatory subpopulation, identified after 3 wk of HFD, to see which of these genes are regulated by interferons and the specific type of IFN it is regulated by. We used the following search conditions: Interferome type: Any, Interferome subtype: Any, Treatments Concentration: Any, Treatment Time: Any, Vivo/Vitro: Any, Species: Mus musculus, System: Any, Organ: Any, Cell: Any, Cell line: Any, Normal/Abnormal: Any, Fold Change Up: 2.0, Fold Change Down: 2.0. Gene symbols were submitted.

### Statistical analysis

The relevant statistical analysis performed in the single-cell analysis is provided in each section of the section “Single Cell Multiome ATAC + Gene expression sequencing Analysis” and “In vivo validation of high inflammatory response β-cells.” All *P*-values were adjusted for false discovery rate (FDR) using the Benjamini and Hochberg method when the number of tests exceeded 10. Unless otherwise stated, statistical tests were performed using the nonparametric Wilcoxon test to compare two means. Significance levels were indicated as follows: *: *P*-value ≤0.05. Boxplots depict the first and third quartiles as the lower and upper bounds of the box, with a band inside the box showing the median value; whiskers represent 1.5 times the interquartile range. Point and line plots indicate mean ± SD (standard deviation).

## Supplementary Material

Reviewer comments

## Data Availability

Further information and requests for resources and reagents should be directed to and will be fulfilled by the lead contact Jesper Grud Skat Madsen (jgsm@bmb.sdu.dk). Raw and processed single-nucleus Multiome ATAC + gene expression sequencing data is available at NCBI GEO under accession number GSE293515. Imaging data are available at the BioImage Archive under accession number S-BIAD3991. All code used in this paper is available on Github: Phenotypic analysis (https://github.com/madsen-lab/islets_multiome_phenotyping), and Single-cell Multiome ATAC + Gene expression sequencing analysis (https://github.com/madsen-lab/islets_multiome_analysis). Access to the public data used in this study has been described in the method section.
