# Supplementary material for: Single nucleus multiomics reveals an early inflammatory response to high-fat diet in mouse islets
Source: Life Sci Alliance. 2026 Sep 1;9(11):e202603840. doi: 10.26508/lsa.202603840 (PMC13534335; doi:10.26508/lsa.202603840)
Supplement: Supplementary file 1 [file LSA-2026-03840_review_history.pdf]

Single Nucleus Multiomics Reveals an Early Inflammatory Response to High-Fat Diet in Mouse Islets

Isabell Ernst, Lasse Lehtonen, Freja Nielsen, Morten Ebbesen, Sille Nilsson, Ann-Britt Marcher, Susanne Mandrup, and Jesper Madsen
DOI: https://doi.org/10.26508/lsa.202603840

Corresponding author(s): Jesper Madsen, University of Southern Denmark and Susanne Mandrup, Univ. of Southern Denmark

Review Timeline:

Submission Date:	2026-07-08
Editorial Decision:	2026-07-13
Revision Received:	2026-08-17
Editorial Decision:	2026-08-19
Revision Received:	2026-08-21
Accepted:	2026-08-21

Scientific Editor: Tim Fessenden

Transaction Report:

(Note: With the exception of the correction of typographical or spelling errors that could be a source of ambiguity, letters and reports are not edited. The original formatting of letters and referee reports may not be reflected in this compilation.)

Please note that the manuscript was previously reviewed at another journal and the reports were taken into account in the decision-making process at *Life Science Alliance*.

Reviews

Referee #1 Review

Report for Author:
The authors have used single nucleus multiomics to address the question of pancreatic beta cell compensation and decompensation in type 2 diabetes using mice fed a high fat diet for 1 and 3 weeks. The rationale of the work is strong, the authors have great expertise in the technologies and bioinformatics. They observe thousands of changes and they work suggest that inflammation pathway related to TNF and interferon may play a role in ß-cell compensation and failure together with possible additional mechanisms.
The work is overall well done, but is primarily descriptive, only omics work and unfortunately not conclusive. It provides interesting data for subsequent mechanistic work and this is generally the goal of omics.
Below are comments that the authors may find useful
Major
1) HFD mice show tremendous heterogeneity in response to HFD. Some mice barely respond, others dramatically respond in term of body weight gain and most are scattered 'in the middle". Usually one needs minimum 8 mice per group to compensate for this problem. There are only two mice per time and HFD so the problem is evident as we do not know if the mice can really be compared among groups.
2) There are no experiments done to prove that what is suggested may be true like western blots of key mRNA that vary, secretion experiments, insulin content, UPR measures, metabolic studies etc
3) Insulin secretion is key but not directly measured for example during IpGTT. SPINA-Gß is used as surrogate but is it a good index and what is it precisely should be better explained.
4) Very high glucose (22 mM) and palmitate in vitro expts in human islets and the HFD mice cannot be really compared as the glycemia is mice is very slightly increased and it is known that FFA are not changed in HFD mice
5) The goal here in particular is to have hints about beta cell decompensation. However Figure 1 does not show this as overall all panels show similar values at 1 and 3 weeks and even insulin resistance as determined by ITT is similar to controls at 3 weeks which is unanticipated.

Referee #2 Review

Report for Author:
This manuscript by Isabell Victoria Strandby Ernst et al. employs a single-cell multiomic approach to investigate the response of mouse pancreatic islets to short-term high-fat diet (HFD) exposure. The authors demonstrate that pancreatic β-cells are the most profoundly affected islet cell type under this metabolic stress. Notably, eRegulome analysis reveals a type I interferon response signature in the stressed β-cells, transcriptionally regulated by interferon-responsive factors including Irf2, Irf9, Stat1, and Stat2. The study further shows that this inflammatory program is heterogeneously activated within the β-cell population, with a subset of cells exhibiting a markedly stronger response. Interestingly, similar heterogeneity in interferon responsiveness is also observed in β-cells from human with type 1 and type 2 diabetes. This study provides a valuable resource for characterizing β-cell responses to metabolic stress, and many of the analyses are carefully and elegantly performed. However, the current scope of data and analysis is limited and does not fully support the major conclusions drawn. As such, the manuscript, in its present form, may not meet the standard for publication in The EMBO Journal.
Specifically, I would suggest the authors to address the following major concerns:
1. The first two figures do not present novel or major findings. Figure 1 primarily confirms that short-term HFD feeding induces expected metabolic phenotypes-results that have been consistently reported in prior studies using this model. Figure 2 provides a basic classification of islet cell types, serving to validate the single-cell assay. However, similar islet cell profiles have already been published extensively, including in human datasets using more advanced single-cell technologies (e.g., PMID: 39472434). While these figures are important as quality control for the experimental design, they do not provide new biological insight and would be more appropriately placed in the supplementary materials. As a result, only three main figures remain that present substantive biological findings, which limits the overall impact and suitability of the manuscript for publication in The EMBO Journal.
2. In Figure 3A, the cell type prioritization score for β-cells appears only marginally higher than for other islet cell types, and it is unclear whether this difference is statistically significant. The authors acknowledge that the higher number of β-cells may introduce bias into this analysis and attempt to address this by correlating prioritization scores with cell numbers across cell types (Figure S3A). However, this correlation analysis does not fully resolve the concern, as the prioritization scores differ only modestly across cell types despite large disparities in cell numbers. To more

rigorously assess whether β-cells are genuinely more responsive to HFD, I recommend downsampling the β-cell population to match the cell numbers of each of the other islet cell types and reanalyzing prioritization scores. They should show that with comparable cell number, β-cells still have the highest prioritization score.
3. The findings in Figure 3E are intriguing and raise important questions about the relevance of in vitro stress models. As the authors state, "Glucose (22 mM), palmitate (0.5 mM), or glucose and palmitate together did not induce gene programs similar to any of the HFD-induced clusters in murine islets in this study, suggesting that glucose and palmitate, when added ex vivo, cannot recapitulate the effects of short-term HFD." However, additional analyses are needed to support this conclusion more rigorously. For example, do any of the differentially expressed genes induced by glucose and/or palmitate overlap with those observed in HFD-treated β-cells? If so, what proportion of the HFD-responsive genes are captured by these in vitro models? Performing gene set enrichment analysis (GSEA) using in vitro-induced gene sets against HFD-ranked gene lists would provide further insight into the degree of similarity between in vitro and in vivo responses.
4. Similarly, it is not surprising that in vitro cytokine treatment induces an inflammatory gene module, corresponding to cluster 2 in Figure 3D. However, other gene clusters that exhibit dynamic regulation during HFD exposure are not recapitulated by cytokine treatment, suggesting that this model captures only a limited subset of the in vivo transcriptional response. It is also possible that glucose/palmitate treatment induces a partial HFD-like signature that was not detected under the current clustering resolution. Increasing the number of clusters in the fuzzy clustering analysis may uncover additional overlaps between in vitro and in vivo conditions. To more comprehensively compare β-cell responses to HFD feeding and in vitro stress exposure, the authors could perform a principal component analysis (PCA) or correlation analysis using pseudobulk β-cell profiles from both their dataset and published in vitro-treated islet datasets.
In summary, the comparison between in vivo and in vitro stress responses addresses an important and timely question. However, the current analysis remains superficial. A more in-depth investigation could yield meaningful insights and significantly enhance the impact of the study.
5. The SCENIC+ and eRegulon analyses highlight important transcriptional regulators involved in the β-cell response to HFD, particularly type I interferon-related transcription factors. To strengthen these findings, it would be valuable to experimentally validate one or more key regulators, such as Stat2. For example, in vitro knockdown of Stat2 in islets isolated from HFD-treated mice or in islets exposed to type I interferons could help determine the causal role of this factor in driving the observed gene expression programs.
6. The observed heterogeneity in β-cell responsiveness to inflammation represents a major finding of this study. However, the current analysis is primarily descriptive and would be significantly strengthened by experimental validation. For example, fluorescence in situ hybridization (FISH) or immunostaining for key inflammatory markers could be employed to visualize and confirm the presence of β-cell subpopulations with high or low interferon responses within intact islets. Such spatial validation could provide additional insights not accessible through single-cell alone. For instance, it would clarify whether these β-cell states represent distinct subtypes within the same islet or reflect inter-islet variability (e.g., a normal islet versus one undergoing insulitis). Furthermore, if both β-cell populations coexist within the same islet, it would be valuable to determine whether the more inflamed β-cells are preferentially associated with immune cells, blood vessels, or localized closer to the islet periphery.
7. The claims regarding β-cell heterogeneity could be further strengthened through more in-depth computational analysis. In the current study, β-cell subtypes are defined using a simple threshold on inflammatory response gene activity scores. This binary classification may overlook more nuanced transcriptional variation. It would be informative to visualize these putative subpopulations on the UMAP embedding and assess whether they occupy distinct regions. Additionally, the use of more standard unsupervised clustering approaches (e.g., Leiden or Louvain algorithms) could help determine whether the subtypes emerge naturally from the data. If conventional clustering methods are insufficient, more advanced machine learning approaches, such as those described in PMID: 37231096 and 38677282, may provide greater sensitivity in identifying subtle but biologically meaningful cell states. Similar approaches should be applied to the identification of corresponding human β-cell subpopulations in Figure 5E.
8. In Figure 5I, β-cell heterogeneity is defined using a threshold based on unfolded protein response (UPR) gene activity. Similar to the approach used for classifying inflammatory subtypes, this method relies on a simple cutoff, which may not reliably capture the full spectrum of biological variability. Given the high gene dropout rate typical of 10x Genomics single-cell datasets, such threshold-based classifications can be sensitive to noise and prone to misclassification. The authors should consider applying more robust approaches-such as unsupervised clustering or advanced modeling techniques that better account for technical variability-to define β-cell subpopulations. Furthermore, it would be informative to assess the degree of overlap between the UPR-based and inflammation-based classifications. Do these represent distinct β-cell states, or is there substantial convergence between the two types of heterogeneity?
And here are a few minor issues:

9. In the metabolic studies in Figure 1, β-cell function is measured as insulin secretory capacity using SPINA (Structure Parameter Inference Approach)-Carb assay. According to the method, this assay relies on steady-state fasting concentrations of serum insulin (pmol/L) and blood glucose (mmol/L), which does not provide any information about glucose stimulated insulin secretion potential. Standard glucose stimulated insulin secretion assays (both in vivo and in vitro) should be employed as more accurate measurements of β-cell function.
10. Figure 3C, color code not labeled.
11. "From each dataset, we extracted the β-cells and calculated gene module activity scores for each of the five clusters of DEGs (Figure 3C and Figure S3C)." This should be Figure 3E and Figure S3D.
12. Methods section explaining Figure 3E indicated that "Differentially expressed genes were identified between treated and untreated β-cells following 24 or 72 hours of treatment using pseudobulked counts, and a DESeq2 paired Wald test as described in the section "Differential gene expression analysis... Subsequently, these genes were then used to compute gene module activity scores for each β-cell, following the procedure outlined in the section "Gene Module Activity". Figure 3E seems to use gene clusters in Figure 3D for module activity calculation, is this correct?
13. In Figure 4C, the ordering of phenotypic traits appears unclear. For example, glucose-related traits are presented in a non-chronological order (e.g., 4-week, 10-week, 8-week, then 6-week), and are interspersed with triglyceride traits. Reordering the traits by type and time point would improve clarity and may also help reveal temporal dynamics in the activation of different eRegulons.
14. It is unclear whether Figure 5E represents the percentage of highly inflamed β-cells or the average inflammatory gene activity score. If the figure displays the percentage of inflamed cells, then the figure title and legend need to be corrected to reflect this. If it instead shows the average gene activity score, then the corresponding description in the main text appears to be inaccurate. The authors should clarify this discrepancy to ensure consistency between the figure, legend, and text.

In summary, I would suggest the authors to focus on the following aspects for improvements:

1. Expand the Dataset and Analysis

To meet the expectations of a high-impact journal, the manuscript would benefit from additional data and deeper biological insights beyond the current figures.

2. Include Experimental Validation

Key conclusions should be supported by experimental validation using orthogonal methods to strengthen the overall claims.

3. Strengthen Computational Analyses

Major findings would be more convincing if supported by more robust and comprehensive computational analyses.

July 13, 2026

Re: Life Science Alliance manuscript #LSA-2026-03840-T

Jesper GS Madsen
University of Southern Denmark
Department of Biochemistry and Molecular Biology
Denmark

Dear Dr. Madsen,

Thank you for transferring your manuscript entitled "Single Nucleus Multiome Analysis Reveals Early Inflammatory Response to High-Fat Diet in Mouse Pancreatic Islets" to Life Science Alliance along with the original reviewer reports. In accordance with our offer to publish this work, kindly provide a revised manuscript with the following changes:

- Confirm the central claim of heterogeneity in inflammatory responses of islet cells in a manner of your choosing (Reviewer 1 point 2, Reviewer 2 point 6).
- Examine heterogeneity in inflammatory response in greater detail using existing data (Reviewer 2 point 7).
- Discuss the potential limitations of these conclusions given the low n used for transcriptomic analyses, especially LFD at 3 weeks (n = 1), as noted by Reviewer 1 point 1.
- Optional: consider extending the analysis in Fig 3E to dissect shared gene expression profiles between in vitro and in vivo perturbations (Reviewer 2 point 3).

As noted in our prior correspondence, a revised manuscript must be accompanied by a rebuttal letter that addressed all reviewer points in some form. However, additional changes to the manuscript beyond those noted above are left to your discretion. I would be happy to discuss the revision in more detail via email or phone/videoconferencing. Please let me know which option you prefer, if any.

To upload the revised version of your manuscript, please log in to your account: https://lsa.msubmit.net/cgi-bin/main.plex

You will be guided to complete the submission of your revised manuscript and to fill in all necessary information. Please get in touch in case you do not know or remember your login name.

While you are revising your manuscript, please also attend to the below editorial points to help expedite the publication of your manuscript. Please direct any editorial questions to the journal office.

Thank you for this interesting contribution to Life Science Alliance. We are looking forward to receiving your revised manuscript.

Sincerely,

Tim Fessenden
Scientific Editor
Life Science Alliance

A. THESE ITEMS ARE REQUIRED FOR REVISIONS

-- A letter addressing the reviewers' comments point by point.

-- An editable version of the final text (.DOC or .DOCX) is needed for copyediting (no PDFs).

-- High-resolution figure, supplementary figure and video files uploaded as individual files: See our detailed guidelines for preparing your production-ready images, https://www.life-science-alliance.org/authors

-- Summary blurb (enter in submission system): A short text summarizing in a single sentence the study (max. 200 characters including spaces). This text is used in conjunction with the titles of papers, hence should be informative and complementary to the title and running title. It should describe the context and significance of the findings for a general readership; it should be written in the present tense and refer to the work in the third person. Author names should not be mentioned.

-- By submitting a revision, you attest that you are aware of our payment policies found here: https://www.life-science-alliance.org/copyright-license-fee

B. MANUSCRIPT ORGANIZATION AND FORMATTING:

Full guidelines are available on our Instructions for Authors page, https://www.life-science-alliance.org/authors

We encourage our authors to provide original source data, particularly uncropped/-processed electrophoretic blots and spreadsheets for the main figures of the manuscript. If you would like to add source data, we would welcome one PDF/Excel-file per figure for this information. These files will be linked as supplementary "Source Data" files.

IMPORTANT: It is Life Science Alliance policy that if requested, original data images must be made available. Failure to provide original images upon request will result in unavoidable delays in publication. Please ensure that you have access to all original microscopy and blot data images before submitting your revision.

Point-by-point response to editorial comments

Comment 1: Confirm the central claim of heterogeneity in inflammatory responses of islet cells in a manner of your choosing (Reviewer 1 point 2, Reviewer 2 point 6).

RESPONSE: To experimentally confirm that this subtype of high inflammatory response β-cells exists, we performed high-throughput immunofluorescence staining of pancreatic tissue from mice fed either LFD or HFD for 3 weeks (n = 4 per diet, two slides per mice, 2 tissues per slide). We stained tissue sections for insulin (to identify β-cells and islets), STAT1 (as a marker of high inflammatory β-cells), and DAPI (to mark cell nuclei) (**NEW Figure 5F**). We used a custom semi-automated imaging and analysis pipeline which allowed us to capture islets across the entire tissue section. Segmentation of the insulin and DAPI staining allowed us to capture 64-149 islets and 6,606-15,670 individual β-cells per mouse. We measured the mean pixel intensity of STAT1 within individual insulin-positive (β-) cells and classified the cells with the highest STAT1 levels as "STAT1 high" β-cells. Consistent with our original hypothesis, HFD-fed mice had a significantly higher proportion of these STAT1 high β-cells compared to LFD-fed mice ($p < 0.05$) (**NEW Figure 5G**), confirming that in intact tissue, and at scale, that HFD increases the number of high inflammatory response β-cells. Finally, we examined whether STAT1 high cells were located in a particular spatial location of the islet. We found that β-cells at the outer edge (periphery) of the islet had significantly higher odds of being STAT high compared to cells in the core ($p < 0.05$) (**NEW Figure 5J**).

Comment 2: Examine heterogeneity in inflammatory response in greater detail using existing data (Reviewer 2 point 7).,

RESPONSE: When we visualize the high inflammatory β-cell subtypes on the UMAP embedding, we see that they occupy a distinct region (**NEW Figure S5E**). This section of the UMAP embedding is also enriched for gene expression of Stat1 and Stat2 (**NEW Figure S5D**). To address concerns about the binary classification approach, we performed unsupervised clustering on the β-cell subset. Louvain clustering (resolution 0.2) on the weighted nearest neighbor graph identified 6 distinct populations. Cluster 3 was comprised of 60.1% high inflammatory cells (Identified using thresholding), compared to 0.6-9.1% in other clusters. Of the 58 upregulated genes in cluster 3, 18 were TNF and IFNβ target genes, with 8 among the top 10 marker genes ranked by percentage expressed, FDR, and log2 fold-change (**NEW Figure S5G**). This demonstrates that the inflammatory β-cell state emerges organically from the transcriptomic and chromatin landscape supporting our initial classification.

Comment 3: Discuss the potential limitations of these conclusions given the low n used for transcriptomic analyses, especially LFD at 3 weeks (n = 1), as noted by Reviewer 1 point 1.

RESPONSE: We have added a discussion of the limitations of our work to the manuscript in the end of the Discussion.

Comment 4: consider extending the analysis in Fig 3E to dissect shared gene expression profiles between in vitro and in vivo perturbations (Reviewer 2 point 3).

RESPONSE: We have added GSEA as suggested by the reviewer (**NEW Figure S3E**), which supports the existing analyses.

Point-by-point response to reviewer #1

We would like to thank the reviewer for the detailed review of our work. The changes implemented in the manuscript based on this feedback have improved the overall quality of the manuscript. Please find below a point-by-point response to each raised comment.

Comment 1: HFD mice show tremendous heterogeneity in response to HFD. Some mice barely respond, others dramatically respond in term of body weight gain and most are scattered 'in the middle". Usually, one needs minimum 8 mice per group to compensate for this problem. There are only two mice per time and HFD so the problem is evident as we do not know if the mice can really be compared among groups.

RESPONSE: We acknowledge the reviewer's concern that the N for the multi-omics experiment is rather small. That said, two lines of evidence support the reliability of our sequencing data despite the small N: first, outlier analysis based on absolute weight and weight gain of the mice, showed that the sequenced mice are comparable to the broader cohort rather than skewed toward one extreme; second, principal component analysis performed separately for each islet cell type showed separation by dietary condition along PC1 (53–77% of explained variance), with additional separation along PC2 (13–19%), indicating that the diet effect is consistent and detectable above inter-individual noise. We have added discussion about the implications of small N in the Discussion in the manuscript on page 12.

Comment 2: There are no experiments done to prove that what is suggested may be true like western blots of key mRNA that vary, secretion experiments, insulin content, UPR measures, metabolic studies etc

RESPONSE: We agree that direct functional validation of the transcriptional changes identified in this study would further strengthen the mechanistic interpretation of our findings. The primary objective of this study was to characterize the early islet response to short-term HFD exposure in terms of changes in the transcriptome and epigenome of individual cells. Our observations led us to generate and present hypotheses regarding the regulatory mechanisms involved in early β-cell compensation / decompensation. We have partly supported these hypotheses through physiological characterization of the animals, including measurements of fasting glucose, fasting insulin, glucose tolerance, insulin sensitivity, and model-based estimates of β-cell secretory capacity. We have now also experimentally confirmed the existence of the high inflammatory response β-cell subtype. We have performed high-throughput immunofluorescence staining of pancreatic tissue from mice fed either LFD or HFD for 3 weeks (n = 4 per diet, two slides per mice, 2 tissues per slide). We stained tissue sections for insulin (to identify β-cells and islets), STAT1 (as a marker of high inflammatory β-cells), and DAPI (to mark cell nuclei) (**NEW Figure 5F**). We used a custom semi-automated imaging and analysis pipeline which allowed us to capture islets across the entire tissue section. Segmentation of the insulin and DAPI staining allowed us to capture 64-149 islets and 6,606-15,670 individual β-cells per mouse. We measured the mean pixel

intensity of STAT1 within individual insulin-positive (β-) cells and classified the cells with the highest STAT1 levels as "STAT1 high" β-cells. Consistent with our original hypothesis, HFD-fed mice had a significantly higher proportion of these STAT1 high β-cells compared to LFD-fed mice ($p < 0.05$) (**NEW Figure 5G**), confirming that in intact tissue, and at scale, that HFD increases the number of high inflammatory response β-cells. Finally, we examined whether STAT1 high cells were located in a particular spatial location of the islet. We found that β-cells at the outer edge (periphery) of the islet had significantly higher odds of being STAT high compared to cells in the core ($p < 0.05$) (**NEW Figure 5J**). Nevertheless, we agree that the functional consequences of the identified transcriptional programs remain to be established experimentally. We therefore consider the present work primarily as a resource and hypothesis-generating study that identifies candidate pathways, transcription factors, and cellular states for future mechanistic investigation.

Comment 3: Insulin secretion is key but not directly measured for example during IpGTT. SPINA-Gβ is used as surrogate but is it a good index and what is it precisely should be better explained.

RESPONSE: SPINA-Gβ is a model-derived index of β-cell secretory capacity, calculated from fasting glucose and insulin using the SPINA-Carb model (a non-linear, physiologically based model of the glucose-insulin feedback loop, unlike the linear approximation underlying HOMA-B), providing a steady-state estimate of maximal secretory capacity without requiring a dynamic stimulation test. We have provided more detail on this measure in manuscript on page 3.

Furthermore, to show that SPINA-Gβ can be used as a surrogate for directly measured insulin secretion, we calculated it from fasting glucose/insulin values reported in an independent, published dataset[1] of mice on chow diet (CD) vs. HFD, and compared it against that study's own directly measured, glucose-stimulated insulin secretion (GSIS AUC) at 2 days and stimulation index (secretion at low (2.5 mM) vs. high (16.7 mM) glucose) at 4 weeks (**Figure 1 in this document**). In this cohort, SPINA-Gβ was elevated in the HFD groups compared to controls, at both timepoints, this was in concordance with the GSIS and stimulation index which was also elevated in the HFD group at both time points, demonstrating that SPINA-Gβ reliably can be used as a surrogate of β-cell secretory capacity.

[Figure removed by editorial staff per authors' request]

Comment 4: Very high glucose (22 mM) and palmitate in vitro expts in human islets and the HFD mice cannot be really compared as the glycemia is mice is very slightly increased and it is known that FFA are not changed in HFD mice

RESPONSE: We agree that the conditions used in the human *ex vivo* studies, particularly 22 mM glucose and 0.5 mM palmitate, do not directly recapitulate the physiological state of our HFD-fed mice. Our intention was not to establish a direct physiological equivalence between these experimental systems, but rather to use available human β-cell perturbation datasets as a reference to determine whether the transcriptional programs observed in HFD-exposed mouse islets more closely resemble responses to nutrient stress or inflammatory stress. Within this context, we found that the HFD-associated gene expression programs showed limited similarity to those induced by high glucose and/or palmitate, whereas stronger similarities were observed with those induced by inflammatory cytokine exposure.

Comment 5: The goal here in particular is to have hints about beta cell decompensation. However Figure 1 does not show this as overall all panels show similar values at 1 and 3 weeks and even insulin resistance as determined by ITT is similar to controls at 3 weeks which is unanticipated.

RESPONSE: We respectfully disagree that Figure 1 fails to demonstrate an effect of HFD. HFD-fed mice display significantly increased fasting glucose, fasting insulin, body weight gain, and impaired glucose tolerance, and onset of insulin resistance, relative to LFD controls, indicating the rapid development of metabolic dysfunction and increased insulin demand. These observations are consistent with previous reports using short-term HFD feeding models. We acknowledge, however, that the physiological measurements do not demonstrate a dramatic deterioration between the one- and three-week HFD time points.

Our interpretation was not that overt β-cell failure develops within this short time frame, but rather that the three-week time point displays molecular features that may precede β-cell decompensation. Specifically, the expansion of the inflammatory β-cell population, persistent inflammatory gene programs, and incomplete restoration of β-cell identity gene expression in this subset suggest the emergence of a transcriptional state associated with impaired adaptation.

References

1. Lin, H., Suzuki, K., Smith, N., Li, X., Nalbach, L., Fuentes, S., Spigelman, A.F., Dai, X.Q., Bautista, A., Ferdaoussi, M., et al. (2024). A role and mechanism for redox sensing by SENP1 in β-cell responses to high fat feeding. Nat Commun *15*, 334. 10.1038/s41467-023-44589-x.

Point-by-point response to reviewer #2

We would like to thank the reviewer for the detailed review of our work. The changes implemented in the manuscript based on this feedback have improved the overall quality of the manuscript. Please find below a point-by-point response to each raised comment.

Comment 1: The first two figures do not present novel or major findings. Figure 1 primarily confirms that short-term HFD feeding induces expected metabolic phenotypes-results that have been consistently reported in prior studies using this model. Figure 2 provides a basic classification of islet cell types, serving to validate the single-cell assay. However, similar islet cell profiles have already been published extensively, including in human datasets using more advanced single-cell technologies (e.g., PMID: 39472434). While these figures are important as quality control for the experimental design, they do not provide new biological insight and would be more appropriately placed in the supplementary materials. As a result, only three main figures remain that present substantive biological findings, which limits the overall impact and suitability of the manuscript for publication in The EMBO Journal.

RESPONSE: We agree that Figures 1 and 2 do not represent the primary biological discoveries of the study. We respectfully note that physiological characterization and cell type annotation are standard components of studies using single-cell methods and provide essential context for the biological findings presented in the subsequent figures, hence, we believe these figures are appropriately retained in the main manuscript.

Comment 2: In Figure 3A, the cell type prioritization score for β-cells appears only marginally higher than for other islet cell types, and it is unclear whether this difference is statistically significant. The authors acknowledge that the higher number of β-cells may introduce bias into this analysis and attempt to address this by correlating prioritization scores with cell numbers across cell types (Figure S3A). However, this correlation analysis does not fully resolve the concern, as the prioritization scores differ only modestly across cell types despite large disparities in cell numbers. To more rigorously assess whether β-cells are genuinely more responsive to HFD, I recommend downsampling the β-cell population to match the cell numbers of each of the other islet cell types and reanalyzing prioritization scores. They should show that with comparable cell number, β-cells still have the highest prioritization score.

RESPONSE: We thank the reviewer for this suggestion. The downsampling procedure proposed by the reviewer is already accounting for within the Augur framework. Specifically, Augur estimates prioritization scores using repeated subsampling of a fixed number of cells from each cell type. In our analysis, we used a subsample size of 12 cells per cell type (subsample_size = 12) and 150 independent subsampling iterations (n_subsamples = 150). Therefore, the prioritization scores were not calculated using all β-cells, but rather from repeated equal-sized subsamples across cell types, which minimizes bias arising from differences in cell abundance.

Comment 3: The findings in Figure 3E are intriguing and raise important questions about the relevance of in vitro stress models. As the authors state, "Glucose (22 mM), palmitate (0.5 mM), or glucose and palmitate together did not induce gene programs similar to any of the HFD-induced clusters in murine islets in this study, suggesting that glucose and palmitate, when added ex vivo, cannot recapitulate the effects of short-term HFD." However, additional analyses are needed to support this conclusion more rigorously. For example, do any of the differentially expressed genes induced by glucose and/or palmitate overlap with those observed in HFD-treated β-cells? If so, what proportion of the HFD-responsive genes are captured by these in vitro models? Performing gene set enrichment analysis (GSEA) using in vitro-induced gene sets against HFD-ranked gene lists would provide further insight into the degree of similarity between in vitro and in vivo responses.

RESPONSE: We thank the reviewer for this suggestion. We agree that the conditions used in the human *ex vivo* studies, particularly 22 mM glucose and 0.5 mM palmitate, do not directly recapitulate the physiological state of our HFD-fed mice. Our intention was therefore not to quantify gene-level overlap between these systems, but to use the perturbation datasets as a reference to assess whether the HFD-associated transcriptional programs more closely resemble nutrient or inflammatory stress responses. Using this approach, we observed greater similarity to inflammatory cytokine exposure than to glucose and/or palmitate treatment, which was the specific question addressed by this analysis. We have supported our analysis with GSEA (**NEW Figure S3E**) and find that genes ranked by fold change in expression at three weeks of HFD versus LFD show specific enrichment of gene sets induced by proinflammatory cytokines

Comment 4: Similarly, it is not surprising that in vitro cytokine treatment induces an inflammatory gene module, corresponding to cluster 2 in Figure 3D. However, other gene clusters that exhibit dynamic regulation during HFD exposure are not recapitulated by cytokine treatment, suggesting that this model captures only a limited subset of the in vivo transcriptional response. It is also possible that glucose/palmitate treatment induces a partial HFD-like signature that was not detected under the current clustering resolution. Increasing the number of clusters in the fuzzy clustering analysis may uncover additional overlaps between in vitro and in vivo conditions. To more comprehensively compare β-cell responses to HFD feeding and in vitro stress exposure, the authors could perform a principal component analysis (PCA) or correlation analysis using pseudobulk β-cell profiles from both their dataset and published in vitro-treated islet datasets.

In summary, the comparison between in vivo and in vitro stress responses addresses an important and timely question. However, the current analysis remains superficial. A more in-depth investigation could yield meaningful insights and significantly enhance the impact of the study.

RESPONSE: We thank the reviewer for these suggestions. We agree that the cytokine treatments recapitulate only a subset of the transcriptional changes observed during HFD exposure. However, the purpose of this analysis was not to comprehensively model the HFD response *in vitro*, but rather to determine whether the major HFD-associated gene programs showed greater similarity to nutrient stress or inflammatory stress. Regarding the clustering resolution, the number of fuzzy clusters was not selected arbitrarily. Rather, we determined

the optimal number of clusters using the minimum centroid distance (Dmin) approach implemented in MFuzz, as described in the Methods. We do not believe that PCA, or global correlation analyses would provide a more biologically meaningful comparison for the question addressed here, as the majority of the variance would be attributable to the substantial differences between the datasets, including species, experimental context (*in vivo* versus *ex vivo*), treatment duration, and stimulus intensity. We therefore believe the current analysis is appropriate for supporting the conclusions of the study.

Comment 5: The SCENIC+ and eRegulon analyses highlight important transcriptional regulators involved in the β-cell response to HFD, particularly type I interferon-related transcription factors. To strengthen these findings, it would be valuable to experimentally validate one or more key regulators, such as Stat2. For example, in vitro knockdown of Stat2 in islets isolated from HFD-treated mice or in islets exposed to type I interferons could help determine the causal role of this factor in driving the observed gene expression programs.

RESPONSE: We agree that perturbation experiments targeting key regulators would provide important mechanistic insight into the role of type I interferon signaling in the β-cell response to HFD. However, the primary aim of the present study was to identify candidate regulatory programs and transcription factors associated with early β-cell response to HFD using single-nucleus multi-omics. As such, we view the identification of Stat2 and related regulators as hypothesis-generating findings that provide a foundation for future mechanistic studies. We agree that functional validation of these regulators represents an important direction for future work but is beyond the scope of the current study.

Comment 6: The observed heterogeneity in β-cell responsiveness to inflammation represents a major finding of this study. However, the current analysis is primarily descriptive and would be significantly strengthened by experimental validation. For example, fluorescence in situ hybridization (FISH) or immunostaining for key inflammatory markers could be employed to visualize and confirm the presence of β-cell subpopulations with high or low interferon responses within intact islets. Such spatial validation could provide additional insights not accessible through single-cell alone. For instance, it would clarify whether these β-cell states represent distinct subtypes within the same islet or reflect inter-islet variability (e.g., a normal islet versus one undergoing insulitis). Furthermore, if both β-cell populations coexist within the same islet, it would be valuable to determine whether the more inflamed β-cells are preferentially associated with immune cells, blood vessels, or localized closer to the islet periphery.

RESPONSE: To experimentally confirm that this subtype of high inflammatory response β-cells exists, we performed high-throughput immunofluorescence staining of pancreatic tissue from mice fed either LFD or HFD for 3 weeks (n = 4 per diet, two slides per mice, 2 tissues per slide). We stained tissue sections for insulin (to identify β-cells and islets), STAT1 (as a marker of high inflammatory β-cells), and DAPI (to mark cell nuclei) (**NEW Figure 5F**). We used a custom semi-automated imaging and analysis pipeline which allowed us to capture islets across the entire tissue section. Segmentation of the insulin and DAPI staining allowed us to capture 64-149 islets and 6,606-15,670 individual β-cells per mouse. We measured the mean pixel intensity of STAT1 within individual insulin-positive (β-) cells and classified the cells with the highest STAT1 levels as "STAT1 high" β-cells. Consistent with

our original hypothesis, HFD-fed mice had a significantly higher proportion of these STAT1 high β-cells compared to LFD-fed mice ($p < 0.05$) (**NEW Figure 5G**), confirming that in intact tissue, and at scale, that HFD increases the number of high inflammatory response β-cells. Finally, we examined whether STAT1 high cells were located in a particular spatial location of the islet. We found that β-cells at the outer edge (periphery) of the islet had significantly higher odds of being STAT high compared to cells in the core ($p < 0.05$) (**NEW Figure 5J**).

Comment 7: The claims regarding β-cell heterogeneity could be further strengthened through more in-depth computational analysis. In the current study, β-cell subtypes are defined using a simple threshold on inflammatory response gene activity scores. This binary classification may overlook more nuanced transcriptional variation. It would be informative to visualize these putative subpopulations on the UMAP embedding and assess whether they occupy distinct regions. Additionally, the use of more standard unsupervised clustering approaches (e.g., Leiden or Louvain algorithms) could help determine whether the subtypes emerge naturally from the data. If conventional clustering methods are insufficient, more advanced machine learning approaches, such as those described in PMID: 37231096 and 38677282, may provide greater sensitivity in identifying subtle but biologically meaningful cell states. Similar approaches should be applied to the identification of corresponding human β-cell subpopulations in Figure 5E.

RESPONSE: When we visualize the high inflammatory β-cell subtypes on the UMAP embedding, we see that they occupy a distinct region (**NEW Figure S5E**). This section of the UMAP embedding is also enriched for gene expression of Stat1 and Stat2 (**NEW Figure S5D**). To address concerns about the binary classification approach, we performed unsupervised clustering on the β-cell subset. Louvain clustering (resolution 0.2) on the weighted nearest neighbor graph identified 6 distinct populations. Cluster 3 was comprised of 60.1% high inflammatory cells (Identified using thresholding), compared to 0.6-9.1% in other clusters. Of the 58 upregulated genes in cluster 3, 18 were TNF and IFNβ target genes, with 8 among the top 10 marker genes ranked by percentage expressed, FDR, and log2 fold-change (**NEW Figure S5G**). This demonstrates that the inflammatory β-cell state emerges organically from the transcriptomic and chromatin landscape supporting our initial classification.

Comment 8: In Figure 5I, β-cell heterogeneity is defined using a threshold based on unfolded protein response (UPR) gene activity. Similar to the approach used for classifying inflammatory subtypes, this method relies on a simple cutoff, which may not reliably capture the full spectrum of biological variability. Given the high gene dropout rate typical of 10x Genomics single-cell datasets, such threshold-based classifications can be sensitive to noise and prone to misclassification. The authors should consider applying more robust approaches-such as unsupervised clustering or advanced modeling techniques that better account for technical variability-to define β-cell subpopulations. Furthermore, it would be informative to assess the degree of overlap between the UPR-based and inflammation-based classifications. Do these represent distinct β-cell states, or is there substantial convergence between the two types of heterogeneity?

RESPONSE: We thank the reviewer for this comment. We agree that relying on single genes can be a pitfall in single-cell RNA-seq due to technical dropout. For that reason, we used

module scores, which are composite enrichment-like scores derived from multiple genes. In the case of UPR, the score is calculated from the expression of 38 genes, making the approach robust to noise. UPR activity is a continuum in β-cells making clustering inherently mismatched for the analysis. Instead, we used a thresholding approach, which historically has been used to separate high and low expression cell states in for example FACS. We note that our conclusions do not rely on the existence of discrete UPR-defined cell states, instead we used the same threshold across conditions, and analyzed the relative changes in the proportion of cells with high UPR activity across conditions. This approach makes the analyses more robust to misspecification of the threshold. We have added new analyses quantifying the overlap between the UPR-based and the inflammation-based classifications (**NEW Figure S6H**) and found an overlap that is smaller than expected by random chance. This observation supports the interpretation that the UPR and inflammatory signatures represent related but distinct aspects of the β-cell response to HFD.

Comment 9: In the metabolic studies in Figure 1, β-cell function is measured as insulin secretory capacity using SPINA (Structure Parameter Inference Approach)-Carb assay. According to the method, this assay relies on steady-state fasting concentrations of serum insulin (pmol/L) and blood glucose (mmol/L), which does not provide any information about glucose stimulated insulin secretion potential. Standard glucose stimulated insulin secretion assays (both in vivo and in vitro) should be employed as more accurate measurements of β-cell function.

RESPONSE: SPINA-Gβ is a model-derived index of β-cell secretory capacity, calculated from fasting glucose and insulin using the SPINA-Carb model (a non-linear, physiologically based model of the glucose-insulin feedback loop, unlike the linear approximation underlying HOMA-B), providing a steady-state estimate of maximal secretory capacity without requiring a dynamic stimulation test. We have provided more detail on this measure in manuscript on page 3.

Furthermore, to show that SPINA-Gβ can be used as a surrogate for directly measured insulin secretion, we calculated it from fasting glucose/insulin values reported in an independent, published dataset[1] of mice on chow diet (CD) vs. HFD, and compared it against that study's own directly measured, glucose-stimulated insulin secretion (GSIS AUC) at 2 days and stimulation index (secretion at low (2.5 mM) vs. high (16.7 mM) glucose) at 4 weeks (**Figure 1 in this document**). In this cohort, SPINA-Gβ was elevated in the HFD groups compared to controls, at both timepoints, this was in concordance with the GSIS and stimulation index which was also elevated in the HFD group at both time points, demonstrating that SPINA-Gβ reliably can be used as a surrogate of β-cell secretory capacity.

[Figure removed by editorial staff per authors' request]

Comment 10: Figure 3C, color code not labeled.

RESPONSE: This has been corrected.

Comment 11: "From each dataset, we extracted the β-cells and calculated gene module activity scores for each of the five clusters of DEGs (Figure 3C and Figure S3C)." This should be Figure 3E and Figure S3D.

RESPONSE: This has been corrected.

Comment 12: Methods section explaining Figure 3E indicated that "Differentially expressed genes were identified between treated and untreated β-cells following 24 or 72 hours of treatment using pseudobulked counts, and a DESeq2 paired Wald test as described in the section "Differential gene expression analysis... Subsequently, these genes were then used to compute gene module activity scores for each β-cell, following the procedure outlined in the section "Gene Module Activity". Figure 3E seems to use gene clusters in Figure 3D for module activity calculation, is this correct?

RESPONSE: This has been corrected. Gene clusters in Figure 3D were used for the module activity calculation.

Comment 13: In Figure 4C, the ordering of phenotypic traits appears unclear. For example, glucose-related traits are presented in a non-chronological order (e.g., 4-week, 10-week, 8-week, then 6-week), and are interspersed with triglyceride traits. Reordering the traits by type and time point would improve clarity and may also help reveal temporal dynamics in the activation of different eRegulons.

RESPONSE: Rows and columns were clustered using hierarchical clustering, this has been clarified in the figure legend of Figure 4C.

Comment 14: It is unclear whether Figure 5E represents the percentage of highly inflamed β-cells or the average inflammatory gene activity score. If the figure displays the percentage of inflamed cells, then the figure title and legend need to be corrected to reflect this. If it instead shows the average gene activity score, then the corresponding description in the main text appears to be inaccurate. The authors should clarify this discrepancy to ensure consistency between the figure, legend, and text.

RESPONSE: The figure displays the percentage of highly inflamed β-cells. This has been corrected in the figure title, and figure legend.

References

1. Lin, H., Suzuki, K., Smith, N., Li, X., Nalbach, L., Fuentes, S., Spigelman, A.F., Dai, X.Q., Bautista, A., Ferdaoussi, M., et al. (2024). A role and mechanism for redox sensing by SENP1 in β-cell responses to high fat feeding. Nat Commun *15*, 334. 10.1038/s41467-023-44589-x.

August 19, 2026

RE: Life Science Alliance Manuscript #LSA-2026-03840-TR

Dr. Jesper Grud Skat Madsen
University of Southern Denmark
Department of Biochemistry and Molecular Biology
Campusvej 55
Odense M 5230
Denmark

Dear Dr. Madsen,

Thank you for submitting your revised manuscript entitled "Single Nucleus Multiomics Reveals an Early Inflammatory Response to High-Fat Diet in Mouse Islets". Thank you also for clarifying the provenance of the new Figure 6 in this revision. The reviewer concerns that we felt were necessary to address, concerning inflammatory signaling in islets, among others, have been fully resolved. We would be happy to publish your paper in Life Science Alliance pending final revisions necessary to meet our formatting guidelines.

MANUSCRIPT ORGANIZATION AND FORMATTING:

To avoid unnecessary delays in the acceptance and publication of your paper, please read the following information carefully. Full guidelines are available on our Instructions for Authors page, https://www.life-science-alliance.org/authors

Submission of a paper that does not conform to Life Science Alliance guidelines will delay the acceptance of your manuscript.

-Please upload your supplementary figures as single files; individual figure files should not include captions.
-Please add the X and Bluesky handles of your host institute/organization, as well as your own, and/or one of the authors, in our system.
-Please add an ORCID ID for secondary corresponding author--they should have received instructions on how to do so.
-The Summary should be labeled as 'Abstract'.
-Please consult our manuscript preparation guidelines https://www.life-science-alliance.org/manuscript-prep and make sure your manuscript sections are in the correct order.
-The Method Details section should be labeled as "Materials and Methods".
-Declaration of interests should be labeled as "Conflict of Interest statement".
-Please label the Resource Availability section as "Data Availability". Given that this study did not generate new unique reagents, you may consider removing this subsection. Please also ensure imaging datasets are accessible at EBI.
-Please add your main, supplementary figure, and table legends to the main manuscript text after the references section.
-Contingency Table should be labeled as Table 1 and presented at the end of manuscript file.
-Supplementary Figure S4 contains a single panel; please remove label A from the caption.
-Figure 5: there is a callout for Figure 5J whereas a callout for Figure 5H is missing, please rectify this.
-Please add a callout for Figure 1J and S1C to your main manuscript text.

We encourage our authors to provide original source data, particularly uncropped/-processed electrophoretic blots and spreadsheets for the main figures of the manuscript. If you would like to add source data, we would welcome one PDF/Excel-file per figure for this information. These files will be linked as supplementary "Source Data" files.

It is Life Science Alliance policy that if requested, original data images must be made available to the editors. Failure to provide original images upon request will result in unavoidable delays in publication. Please ensure that you have access to all original data images prior to final submission.

If you are planning a press release on your work, please inform us immediately to allow informing our production team and scheduling a release date.

We welcome submissions of potential cover images for the issue of LSA in which your work would appear. If you have high quality images associated with this work, please feel free to email these, with a caption, to the journal office.

LSA encourages authors to provide a 30-60 second video where the study is briefly explained. These videos will be appear embedded with the manuscript online at Life Science Alliance, and on social media to promote the published paper and authors (for examples, see https://docs.google.com/document/d/1-UWCfbE4pGcDdcgzcmiuJl2XMBJnxKYeqRvLLrLSo8s/edit?

usp=sharing). Corresponding or first-authors are welcome to submit the video. Please submit only one video per manuscript. The video can be emailed to contact@life-science-alliance.org

FINAL FILES:

To upload the final version of your manuscript, please log in to your account: https://lsa.msubmit.net/cgi-bin/main.plex
You will be guided to complete the submission of your revised manuscript and to fill in all necessary information. Please get in touch in case you do not know or remember your login name.

The following items are required for acceptance.

-- An editable version of the final text (.DOC or .DOCX) is needed for copyediting (no PDFs).

-- High-resolution figure, supplementary figure and video files uploaded as individual files: See our detailed guidelines for preparing your production-ready images, https://www.life-science-alliance.org/authors

-- Summary blurb (enter in submission system): A short text summarizing in a single sentence the study (max. 200 characters including spaces). This text is used in conjunction with the titles of papers, hence should be informative and complementary to the title. It should describe the context and significance of the findings for a general readership; it should be written in the present tense and refer to the work in the third person. Author names should not be mentioned.

The license to publish form must be signed before your manuscript can be sent to production. A link to the license to publish form will be available to the corresponding author only. Please take a moment to check your funder requirements.

Reviews, decision letters, and point-by-point responses associated with peer-review at Life Science Alliance will be published, alongside the manuscript. If you do want to opt out of having the reviewer reports and your point-by-point responses displayed, please let us know immediately.

Thank you for your attention to these final processing requirements. Please revise and format the manuscript and upload materials as soon as you are able.

Thank you for this interesting contribution to the literature. We look forward to publishing your paper in Life Science Alliance.

Sincerely,

Tim Fessenden
Scientific Editor
Life Science Alliance

August 21, 2026

RE: Life Science Alliance Manuscript #LSA-2026-03840-TRR

Dr. Jesper Grud Skat Madsen
University of Southern Denmark
Department of Biochemistry and Molecular Biology
Campusvej 55
Odense M 5230
Denmark

Dear Dr. Madsen,

Thank you for submitting your Research Article entitled "Single Nucleus Multiomics Reveals an Early Inflammatory Response to High-Fat Diet in Mouse Islets". It is a pleasure to let you know that your manuscript is now accepted for publication in Life Science Alliance. I am pleased that we could handle this work with the efficiency and I congratulate you and your colleagues on these interesting findings.

The final published version of your manuscript will be deposited by us to PubMed Central upon publication.

Your manuscript will now progress through copyediting and proofing. It is journal policy that authors provide original data upon request.

Your article will publish open access upon publication under a CC-BY license.

Reviews, decision letters, and point-by-point responses associated with peer-review at Life Science Alliance will be published, alongside the manuscript. If you do want to opt out of having the reviewer reports and your point-by-point responses displayed, please let us know immediately.

IMPORTANT: If you will be unreachable at any time, please provide us with the email address of an alternate author. Failure to respond to routine queries may lead to unavoidable delays in publication.

Scheduling details will be available from our production department. You will receive proofs shortly before the publication date. Only essential corrections can be made at the proof stage so if there are any minor final changes you wish to make to the manuscript, please let the journal office know now.

DISTRIBUTION OF MATERIALS:
Authors are required to distribute freely any materials used in experiments published in Life Science Alliance. Authors are encouraged to deposit materials used in their studies to the appropriate repositories for distribution to researchers.

You can contact the journal office with any questions, contact@life-science-alliance.org

Again, congratulations on a very nice paper. I hope you are pleased with how the manuscript was handled editorially. We look forward to future exciting submissions from your lab.

Sincerely,

Tim Fessenden
Scientific Editor
Life Science Alliance
